# Chromosome 3A harbors several pleiotropic and stable drought‐responsive alleles for photosynthetic efficiency selected through wheat breeding

**DOI:** 10.1002/pld3.438

**Published:** 2022-09-02

**Authors:** Ahossi Patrice Koua, Benedict Chijioke Oyiga, Said Dadshani, Salma Benaouda, Mohammad Bahman Sadeqi, Uwe Rascher, Jens Léon, Agim Ballvora

**Affiliations:** ^1^ INRES Pflanzenzüchtung Rheinische Friedrich Wilhelms University Bonn Germany; ^2^ Forschungszentrum Jülich GmbH Jülich Germany; ^3^ Field Lab Campus Klein‐Altendorf University of Bonn Rheinbach Germany

**Keywords:** breeding progress, chlorophyll content, chlorophyll fluorescence, drought, effective quantum yield of photosystem II, GWAS, photosynthesis, wheat

## Abstract

Water deficit is the most severe stress factor in crop production threatening global food security. In this study, we evaluated the genetic variation in photosynthetic traits among 200 wheat cultivars evaluated under drought and rainfed conditions. Significant genotypic, treatments, and their interaction effects were detected for chlorophyll content and chlorophyll fluorescence parameters. Drought stress reduced the effective quantum yield of photosystem II (YII) from the anthesis growth stage on. Leaf chlorophyll content measured at anthesis growth stages was significantly correlated with YII and non‐photochemical quenching under drought conditions, suggesting that high throughput chlorophyll content screening can serve as a good indicator of plant drought tolerance status in wheat. Breeding significantly increased the photosynthetic efficiency as newer released genotypes had higher YII and chlorophyll content than the older ones. GWAS identified a stable drought‐responsive QTL on chromosome 3A for YII, while under rainfed conditions, it detected another QTL on chromosome 7A for chlorophyll content across both growing seasons. Molecular analysis revealed that the associated alleles of AX‐158576783 (515.889 Mbp) on 3A co‐segregates with the NADH‐ubiquinone oxidoreductase (*TraesCS3A02G287600*) gene involved in ATP synthesis coupled electron transport and is proximal to WKRY transcription factor locus. This allele on 3A has been positively selected through breeding and has contributed to increasing the grain yield.

## Highlight

Breeding has aided the selection of multiple pleiotropic and stable drought‐responsive alleles on chromosome 3A associated with improved photosynthetic activity and grain yield under drought stress in winter wheat.

## 1 INTRODUCTION

Frequent drought events are one of the most severe abiotic stress factors in crop production (Basu et al., 2016; Bray, 1997). It is estimated that by 2025, about 65% of the world population will be affected by drought conditions (Hoseinlou et al., 2013; Nezhadahmadi et al., 2013). Agriculture accounts for 80–90% of existing freshwater used by humans, mostly for crop production (Morison et al., 2008). Such use of water resources is considered unsustainable, especially in dry areas under increased pressure and water demand for other purposes (Munjonji et al., 2016; Schlosser et al., 2014). Due to the growing world population, expected to reach 9 billion by 2050, the global water and food production demand will continue to rise. FAO (2009) has predicted an increase of least 50% in food production to meet the future demand. Crop performance under water‐limited conditions is determined by genetic factors controlling yield potential, drought resistance, and water use efficiency (Blum, 2005). Understanding the physiological basis and mechanisms involved in drought resistance is therefore of paramount importance.

Drought resistance can be achieved through several strategies that allow plants to adapt under different episodes of drought stress (Fang & Xiong, 2015). These strategies include drought avoidance (DA), drought tolerance (DT), drought escape (DE), and drought recovery (Fang & Xiong, 2015; Kneebone et al., 1992; Lawlor, 2013; Levitt, 1972; Luo, 2010; Yue et al., 2006). Often, plants combine different mechanisms to withstand water‐deficit stress. Thus, breeding cultivars with high water use efficiency and DT are practical, economical, and have shown promising results to enhance yield under stress conditions (Liu et al., 2010). However, one of the major challenges facing wheat breeders and geneticist is the lack of evaluation of appropriate traits (Araus et al., 1998) and the polygenic nature of traits associated with DT (Peleg et al., 2009). Various research programs aiming at improving wheat drought tolerance included extensive genetic analysis and have contributed to modify the expression of genes involved in stress tolerance. Sometimes, combination of physiological traits and direct selection for higher yield under a range of drought stress scenarios was applied. However, not all expected results have been reached (Langridge & Reynolds, 2021). The cultivars improvement by “physiological breeding” that involves an indirect selection for yield components by measuring physiological parameters such as photosynthesis‐related traits, canopy temperature, and others can be further exploited to improve DT in the new cultivars (Sukumaran et al., 2018).

Photosynthetic capacity and water use efficiency play a major role in wheat growth and productivity under drought conditions (Sallam et al., 2019; Xu et al., 2017). Moreover, Reynolds et al. (2000) have shown that grain yield (GY) is significantly and positively correlated with both photosynthetic rate and stomatal conductance. Makino (2011) and Sánchez et al. (2019) revealed that more than 90% of crop biomass is derived from photosynthetic products. They reported that a genotype with improved photosynthetic activity under stress conditions could produce more biomass, suggesting that improving photosynthetic adaptation to environmental conditions will help to enhance crops biomass production. Drought is a major limiting factor of photosynthesis due to the effect of drought stress on the CO_2_ diffusion as a result of early stomatal closure. Hence, it declines net CO_2_ assimilation rate and restricts crop biomass accumulation (Centritto et al., 2009; Chaves et al., 2003). Drought also reduces both the photochemical efficiency of photosystem II (PSII) considering the decline in chlorophyll pigments and the activity of photosynthetic enzymes (Pandey & Shukla, 2015). Under drought stress conditions, the decrease of stomatal conductance as a result of stomatal closure limits transpirational water loss and aids plants to conserve water status.

Sensor‐based phenotyping has been successfully used to evaluate simultaneously high numbers of genotypes for physiological traits associated with DT in cereals (Ghanem et al., 2015; Sanchez‐Bragado et al., 2014). However, the high costs and the lack of skilled personnel across the globe are major hindrances in using these new technologies in plant sciences and crop breeding. The most used plant physiological phenotyping method for DT under field or controlled conditions is the visual scoring of traits, such as leaf rolling, stay green, and leaf wilting (Sallam et al., 2019, 2018). However, scoring for DT using these traits is time‐consuming and laborious.

To date, few studies have investigated the effect of drought on plant photosynthetic traits across three growth stages among wheat diversity panel that has been cultivated in the past 50 years. Given the importance of photosynthesis in plant growth and development, it is essential to understand the genetic basis influencing this trait. The discovery of new phenomic and photosynthetic traits associated with wheat response to drought stress at genetic and molecular levels will facilitate the development of high yielding drought‐tolerant wheat.

Recent technology developments have led not only to the identification of high numbers of DNA‐markers but also the production of whole‐genome sequence reference of several crops including wheat with its large size of ~17 gigabases (Shi & Ling, 2018; Walkowiak et al., 2020). Genome‐wide association studies (GWAS) have been used in the past decade to dissect the genetic architecture of polygenic traits and identify significant marker‐trait associations (MTAs). Compared with bi‐parental mapping populations, GWAS panels can be developed faster and provide access to a wider range of alleles (Zhu et al., 2008).

In this research, we screened 200 wheat cultivars using several photosynthetic related traits and evaluated their relationship with the aboveground yield and also uncover the quantitative trait loci (QTL) regions associated with photosynthesis under drought and rainfed conditions. Therefore, the objectives of this study were to (1) evaluate the genotypic and drought effects on photosynthesis and transpiration dynamics and unravel at what growth stage drought has highest impact on the final aboveground biomass (yield); (2) to provide information and highlight the positive impact of breeding in the improvement of photosynthetic activity; and (3) uncover drought‐responsive QTL underlying photosynthesis‐related traits.

## 2 MATERIALS AND METHODS

### 2.1 Plant materials, growth conditions, and management

The 200 wheat cultivars diversity panel and the experimental setup used in this study and the weather conditions have been described in a previous study, which highlighted the role played by breeding to enhance yield and its components under drought conditions (Koua et al., 2021). Succinctly, the germplasm was grown under two water regimes in 2017 and 2018 growing seasons at the experimental station of Campus Klein‐Altendorf, University of Bonn (50.61° N, 6.99° E, and 187 m above sea level, Germany). Drought stress was imposed on the plants grown under rainout shelter, while the control plants were grown under rainfed conditions. The drought stress application started at the pre‐booting growth stage, which corresponds to BBCH40 (Biologische Bundesanstalt, Bundessortenamt, und CHemische Industrie [Lancashire et al., 1991]), by stopping the irrigation, and continued until harvesting (BBCH99) as previously described in Koua et al. (2021).

Out of the 200 genotypes evaluated, a subset of 20 genotypes (core set) was selected based on the principal component analysis (PCA) performed with the single nucleotide polymorphisms (SNPs) markers data. This core set that represents the genetic diversity of the entire wheat panel (Figure S1) was phenotyped for photosynthetic traits across three and five growth stages in 2017 and 2018 planting seasons, respectively.

### 2.2 Phenotyping of photosynthesis, agronomic, and grain quality traits

We screened several photosynthetic traits (Tables S1 and S2) including the leaf chlorophyll content (SPAD values) quantified by using the device SPAD‐502Plus (Konica, Minolta, Japan) and the chlorophyll a fluorescence parameters measured using MINI‐PAM II (Mini‐PAM; Effeltrich, Germany) following the manufacturer settings (Walz, 2014). The parameters include the maximum quantum yield of PSII (F_V_/F_M_) measured on dark‐adapted leaves between 0 and 3 am, the effective quantum yield of PSII (YII) measured on the same dates on light‐adapted leaves from 10:00 and 15:00 to minimize higher variation of the prevailing photosynthetic active radiation (PAR), and the non‐photochemical quenching (NPQ). The saturating light pulse for the measurement had a photosynthetic photon flux density (PPFD) intensity of 4,000 mmol m^−2^ s^−1^ and a duration of 800 ms (Rascher et al., 2010). The NPQ was calculated according to the formula provided by Bilger and Björkman (1990) based on dark and light‐adapted leaves measurement. Measurements were done on the core set at various growth stages considering pre‐booting (BBCH30‐39), booting (BBCH40‐49), heading (BBCH50‐59), anthesis (BBCH60‐69), and post‐anthesis (BBCH70‐85). At anthesis, we measured these photosynthetic traits on the 200 genotypes set. Special care was taken throughout all measurements, not to change the ambient conditions of leaves in order to ensure that photosynthesis was in a steady state (Rascher et al., 2010).

Diffusion porometer leaf stomatal conductance (LSCp, mol m^−2^ s^−1^) was measured using AP4‐Porometer (AP4‐Delta‐T Eijelkampt, Giesbech, The Netherlands), while InfraRed Gas Analyzer (IRGA)‐based leaf stomatal conductance (LSCl, mol m^−2^ s^−1^), net photosynthetic rate (A, μmol m^−2^ s^−1^), intercellular CO_2_ concentration (Ci, μmol mol^−1^), transpiration rate (E, mmol m^−2^ s^−1^), and leaf temperature (T, °C) were measured using LI‐6800 (LI‐COR, Lincoln, USA).

We performed visual scorings of developmental traits such as plant health state, homogeneity of growth, leaf rolling, and leaf greenness according to the methods described by Pask et al. (2012). Breeding progress (BP) that has been achieved on agronomic traits including GY, shoot dry weight (SDW), and plant biomass weight (PBW) was described in Koua et al. (2021). In the present study, SNP makers associated to these traits were examined in relation with those underlying photosynthetic traits.

### 2.3 Drought stress tolerance estimation

The stress weighted performance (SWP) status (Saade et al., 2016) was used to identify the genotypes' DT level for GY, SDW, SPAD values, and effective quantum yield of PSII (YII) using the following formula.

(1) $$\mathrm{SWP}=\frac{Y_{S}}{\sqrt{Y_{P}}},$$

where Y_S_ and Y_P_ are the trait phenotypic value under drought and rainfed conditions, respectively.

Thereafter, the genotypes were ranked for each trait from the highest down to the lowest trait's SWP values and were separated into drought‐tolerant and sensitive according to their overall SWP ranking as described by Oyiga et al. (2016).

### 2.4 Statistical analyses of the phenotypic data

A general linear model was used to carry out an analysis of variance (ANOVA) to determine the difference between water regimes (W), genotypes (G), and their interactions (W * G) using R software. Proc Mixed (SAS Institute, 2015) adopting restricted maximum likelihood (REML) was used to compute the best linear unbiased estimates (BLUEs) across each year for each water regime and genotype while errors due to planting positions (row‐and‐column effects) in the field plots were corrected by including “Replication/Row*Column” (Gilmour et al., 1995). These BLUEs were used in downstream analysis including GWAS.

The broad‐sense heritability was calculated within each treatment, using the following equation as described by Gitonga et al. (2014).

(2) $$H_{2}={\left(\sigma_{g}\right)}^{2}/\left[\sigma_{g}^{2}+\sigma_{e}^{2}/r\right],$$

where σ^2^_g_ the variance components due to genotypes, set as random in the mixed model procedure (SAS Institute, 2015), σ^2^_e_ is the residual, and r the number of replicates of each genotype in a treatment.

Narrow sense or marker‐based estimation of heritability (h^2^), which included the kinship‐matrix calculated in TASSEL (available at: http://www.maizegenetics.net/tassel), was estimated using the package “heritability” implemented in R software (Kruijer et al., 2015).

Correlation coefficients (r) for each pair of evaluated traits were done in R using the package *performanceAnalytics*, *while* the *corrplot* package was used to visualize the results. The PCA of photosynthetic related and developmental traits was done with the package *FactoMineR*, and the results were represented in a biplot using the package *factoextra*. To evaluate the representation of a variable on the principal component, the square cosine (Cos^2^) for all variables was plotted using the *corrplot* package.

### 2.5 Evaluation of the BP in evaluated traits

The BP in physiological traits was investigated through linear regression of the trait of interest against the years of release of the genotypes. The adjusted mean values of each genotype under each water regime and growing season were used in the regression analysis. The absolute BP (increase per year) was the slope of the linear regression line between the trait of interest and the year of release (Lichthardt et al., 2020).

### 2.6 SNP genotyping, population structure, and linkage disequilibrium (LD) analysis

The diversity panel was genotyped with 15K Illumina Infinium iSelect chip and with the 135K Affymetrix genotyping array at TraitGenetics GmbH (SGS GmbH Gatersleben, Germany). We used for the genetic analysis, a set of 24,216 SNP markers evenly covering all 21 chromosomes of wheat (Dadshani et al., 2021).

Population structure of the diversity set was determined using 2,769 unlinked SNPs (*r* ^2^ < .7) selected through SNP pruning with Plink software, which adopted the indep‐pairwise algorithm considering a window of 3,500 SNPs that shifted by 350 SNPs forward after each calculations (Purcell, 2010). The analysis of the population structure was done with the 2,769 unlinked SNPs using STRUCTURE v.2.3.4 (Pritchard et al., 2000). The inferred number of sub‐population K tested ranged from 1 to 10, with 10 replications in each test. The true number of K was determined in the structure harvester (Earl, 2012; Evanno et al., 2005). PCA was performed using TASSEL with 24,216 SNP markers set to explore the existing sub‐populations in the panel. Prior imputation of missing SNP values by the mean was done before the PCA analysis.

The LD among SNP pairs within a defined sliding window equal to 10% of the total number of SNPs on the considered chromosome was estimated for A, B, and D genomes in TASSEL. The LD decay was determined by plotting LD (*r* ^2^) values against the distance (megabase pairs) between SNPs on the same chromosome. Thereafter, we deployed a nonlinear regression function (Remington et al., 2001) to fit the trend of LD decay across chromosomes and A, B, and D genomes. The genetic distance corresponding to *r* ^2^ = .1 for each genome and chromosomes was estimated and was considered as the critical distance up to which a QTL could extend.

### 2.7 GWAS and genetic relationship among DT contrasting wheat cultivars

To determine the MTAs, we used the mixed linear model (MLM‐P+K) accounting for population structure calculated by the PCA (P‐matrix) and kinship (K‐matrix), both implemented in software program TASSEL 5 (Yu et al., 2006; Zhang et al., 2010). The association tests were also performed using rrBLUP R package (Endelman, 2011. The GWAS model used is provided below:

(3) Y=Xα+Pβ+Kμ+Ɛ,

where *Y* is the phenotype of a genotype; α and β are unknown vectors containing fixed effects; X the fixed effect of the SNP; P the fixed effect of population structure given by PCA matrix that included the first three components; K the random effect of relative kinship among cultivars; and Ɛ the error term, which is assumed to be normally distributed with mean = 0 and variance δ^2^_e_. GWAS for BP was run with cultivars years of release used as phenotypic values.

To minimize false positives, only congruent significant (*p* < 10^−4^) MTAs in both analyses were retained and reported as significant MTAs in the present study. Thereafter, Benjamini–Hochberg algorithm, which is the false discovery rate (FDR) correction procedure adopted in rrBplup (Mangiafico, 2015), was used to remove false positive at *Q* = .05 using the equation:

(4) $$P=\left(i/m\right)Q$$

where *i* is the SNP P value's rank, from the smaller to the biggest and *m* the total number of tests corresponding to the total number of SNP 24,216 Q the FDR at .05 significance level. The significant (*p* < 10^−4^) SNP loci detected at genetic intervals defined by the chromosomal LD were considered to be in LD (Breseghello & Sorrells, 2006; Pasam & Sharma, 2014) and were grouped into one SNP cluster.

We compared previously reported significant MTAs (*p* = 10^−3^) for agronomic and BP (Koua et al., 2021) with the current ones for the photosynthetic traits measured on the whole panel set at anthesis, to identify SNPs that have pleiotropic effects on these traits and/or are collocating in the same genomic region.

Detected SNP loci associated with BP were subsequently used in a PCA performed in Tassel to analyze the genetic relationships among older and newer released cultivars vis‐à‐vis their tolerance level.

Loci interacting with water regimes were detected using the PROC MIXED procedure in SAS 9.4 (SAS Institute, Cary, NC, USA). The mixed model included the Kinship matrix and PCA matrix calculated in TASSEL as already described. The FDR *Q*‐value cutoffs for accepting highly significant marker * treatment interaction associated with a trait were set at 1 × 10^−4^, and only the first 50 significant SNPs within this threshold for each trait were reported. We also performed a genome‐wide SNP‐SNP epistatic interaction through multi‐locus approach (Afsharyan et al., 2020). The log_10_ *p*‐value cutoff was set at 4 under control to retain at least some significant interactions loci against 15 drought conditions to retain the most significant interactions. The interaction graph was drawn using the package *Circlize* implemented in R (Gu et al., 2014).

### 2.8 Identification of candidate genes in QTL intervals

We searched for candidate genes in the associated QTL intervals of stable or pleiotropic effects on the traits. We took the positions of adjacent SNPs that were not in LD with the SNP–cluster or with the MTAs of interest as the boundary. The searches were performed in the genome assembly of *Triticum aestivum* cv. Chinese Spring (IWGSC et al., 2018) and only high confident (HC) genes were retained.

## 3 RESULTS

### 3.1 Drought stress strongly influenced the dynamics of the photosynthetic traits

**FIGURE 1:**
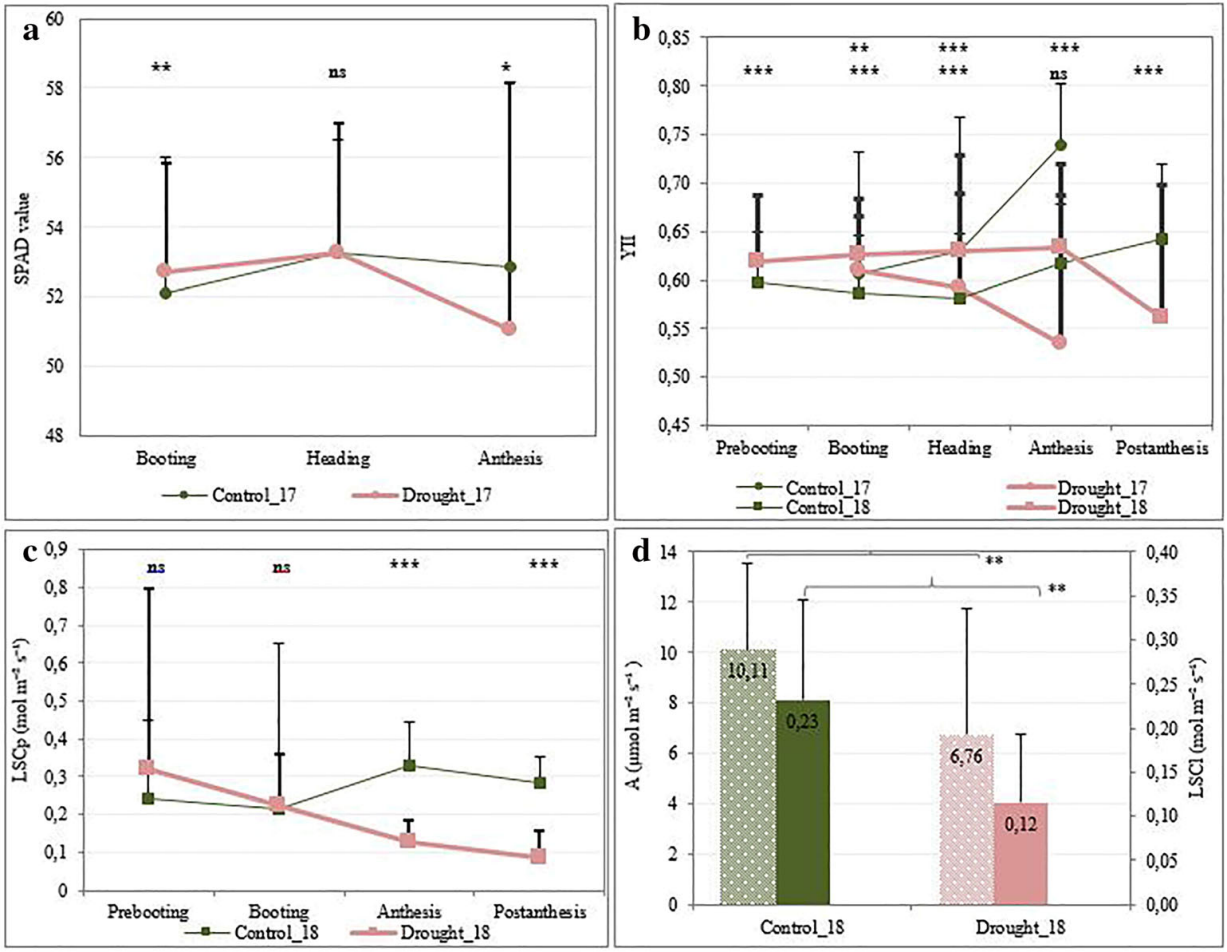
Drought stress effect on the photosynthesis‐related traits across growth stages. (a) Chlorophyll content in 2017; (b) effective quantum yield of PSII under drought (red and large curve) versus control (green and thin curve), in 2017 (circle‐shaped) and 2018 (squared‐shaped). (c) Diffusion porometer‐based leaf stomatal conductance in 2018. (d) Drought stress effect on photosynthetic rate (stars filled barplot) and leaf stomatal conductance (full colored barplot). The significance between both water regimes is given above the graphs and in Table S3. In sub‐panel (b), the first line of significance level is for 2017, whereas the second line is for 2018. The error bars of the curves represent the standard deviation. The thicker error bars are for drought conditions in sub‐panels (a)–(c).

To determine the effect of drought stress on the photosynthetic efficiency, we measured the leaf chlorophyll content and several photosynthesis‐related traits across growth stages under rainfed and drought conditions in 2017 and 2018 growing seasons. ANOVA indicated significant effects of water regimes (W), genotypes (G), and their interactions (W * G) on SPAD values in 2017 and on YII and other fluorescence parameters in 2017 and 2018 and across growth stages (Figure 1; Table S3 and S4). The SPAD values were highest at heading in both control and drought conditions, then it decreased to the lowest value at anthesis under drought conditions. The effective quantum yield of PSII declined by 13.95% from booting to anthesis (in 2017) and by 12.08% from anthesis to post‐anthesis (in 2018), whereas, under control conditions, it increased from heading to anthesis and post‐anthesis in both years (Figure 1a,b). The NPQ that describes plants' protection against excess absorbed light was found to decrease by almost 50% under drought stress in both years (Table S4).

The diffusion‐based leaf stomatal conductance (LSCp) declined from booting by 60.21% to anthesis and by 72.78% to postanthesis under drought stress, while it increased significantly under control conditions (Figure 1c; Table S5). IRGA‐based LSCl, photosynthetic rate (A), transpiration rate (E), and intercellular CO_2_ (Ci) decreased significantly (*p* < .01) by 50.43, 29.53, 66.67, and 8.95% under drought stress at anthesis, respectively (Figure 1d; Table S6).

The standard deviations of traits were higher under drought compared with the rainfed conditions (Figure 1). The coefficient of variations (CV) ranged from 5.95% (for chlorophyll content) at booting to 21.88% (for YII) at heading under rainfed conditions and from 6.20% (chlorophyll content) to 34.75% (YII) under drought conditions. The broad‐sense (*H* ^2^) and narrow‐sense heritability (*h* ^2^) ranged from low values for Fmin under drought (*h* ^2^ = 4.38%) to high values for chlorophyll content under control (*H* ^2^ = 92.57%) (Table S3).

### 3.2 Relationship between photosynthetic traits and DT in wheat

**FIGURE 2:**
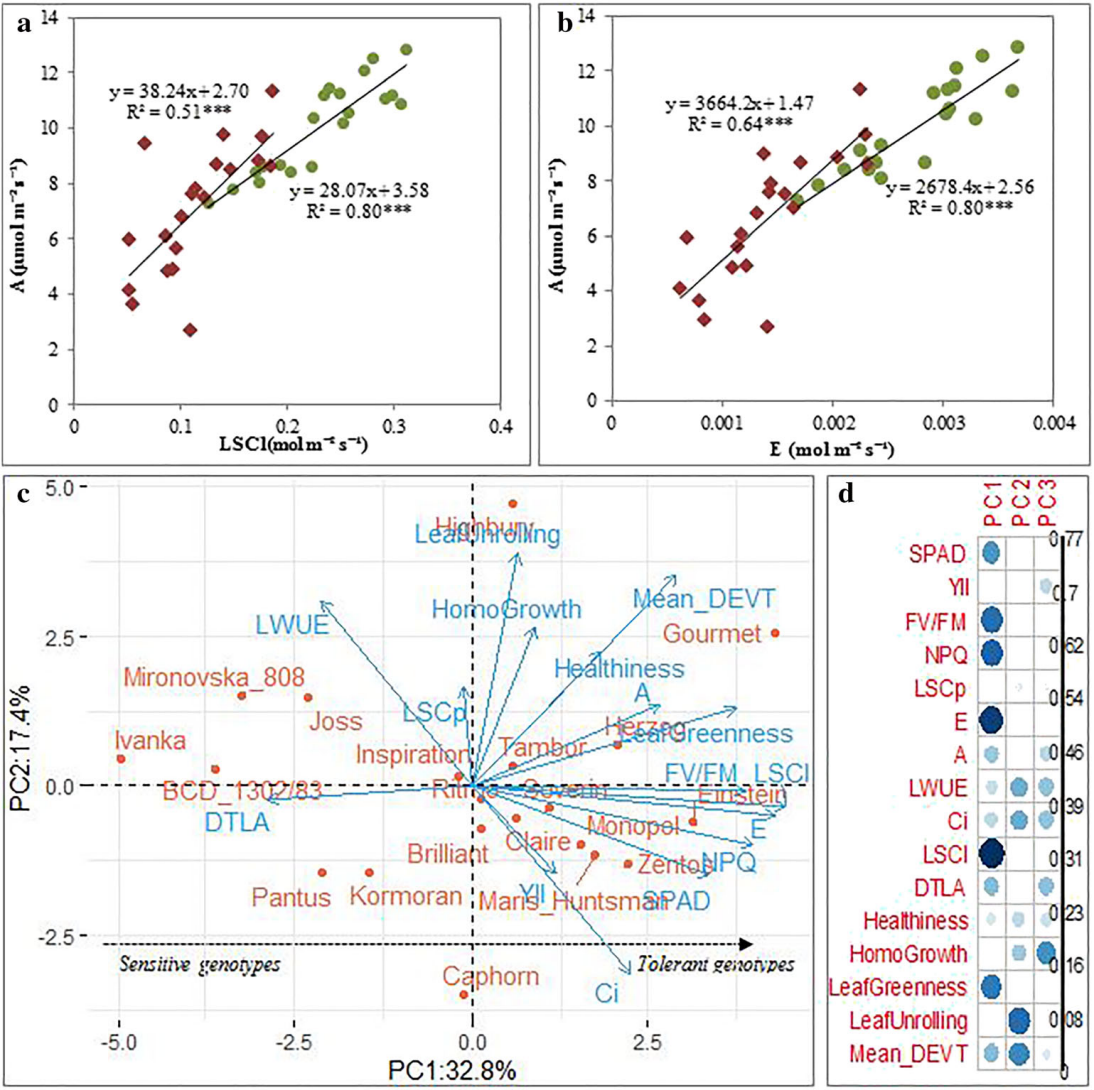
Relationship between photosynthetic rate versus (a) stomatal conductance and (b) transpiration rate after anthesis growth stage in 2018 growing season. Red diamond‐shape and green circle‐shape indicate the genotypes data points under drought and rainfed conditions, respectively. (c) Principal component analysis biplot using 11 photosynthesis‐related variables and 4 visual scored developmental traits under prolonged drought stress conditions. Cosines square of the variables contributing to the newly constructed principal components (d). The size of the circle in sub‐panel (d) indicates the intensity of the variables. The abbreviations of traits name are listed in Table S2.

Pearson coefficient correlation and PCA analysis were performed with the measurements made on the subset at anthesis growth stage to examine the relationship between the photosynthetic traits and developmental traits. Results indicate that SPAD values were positively correlated with NPQ, F_V_/F_M_, and YII under drought and negatively correlated to NPQ under rainfed conditions (Figures 2 and S2). Leaf temperature (DTLA) correlates negatively with transpiration rate (E), photosynthetic rate (A) and IRGA‐based stomatal conductance (LSCl) under both water regimes, and with NPQ and F_V_/F_M_ under drought conditions. Leaf greenness correlated significantly and positively with NPQ and F_V_/F_M_ but was negatively associated with DTLA under drought stress. Photosynthetic rate correlated positively with LSCl and transpiration rate under control and drought stress conditions. The slopes of the regressions of photosynthetic rate versus LSCl and photosynthetic rate versus transpiration rate indicated that one unit increase in LSCl and transpiration rate enhanced the photosynthetic rate more under drought than under control conditions (Figure 2a,b). The first two principal components (PCs) explained 50.2% of the cumulative variance in 11 photosynthetic traits and four developmental traits scored under drought stress conditions (Figure 2c,d). PC1 constituted a gradient of DT oriented from the left with sensitive genotypes (Ivanka, BCD_1302/83, and Mironovska_808) towards the right side of the biplot with tolerant genotypes (Einstein, Gourmet, and Zentos). Comparison of PCA biplot under drought and control conditions revealed that genotypes could show different performance across both water regimes (Figure S3).

### 3.3 Improved photosynthesis at post‐anthesis increases the GY performance under drought stress

**FIGURE 3:**
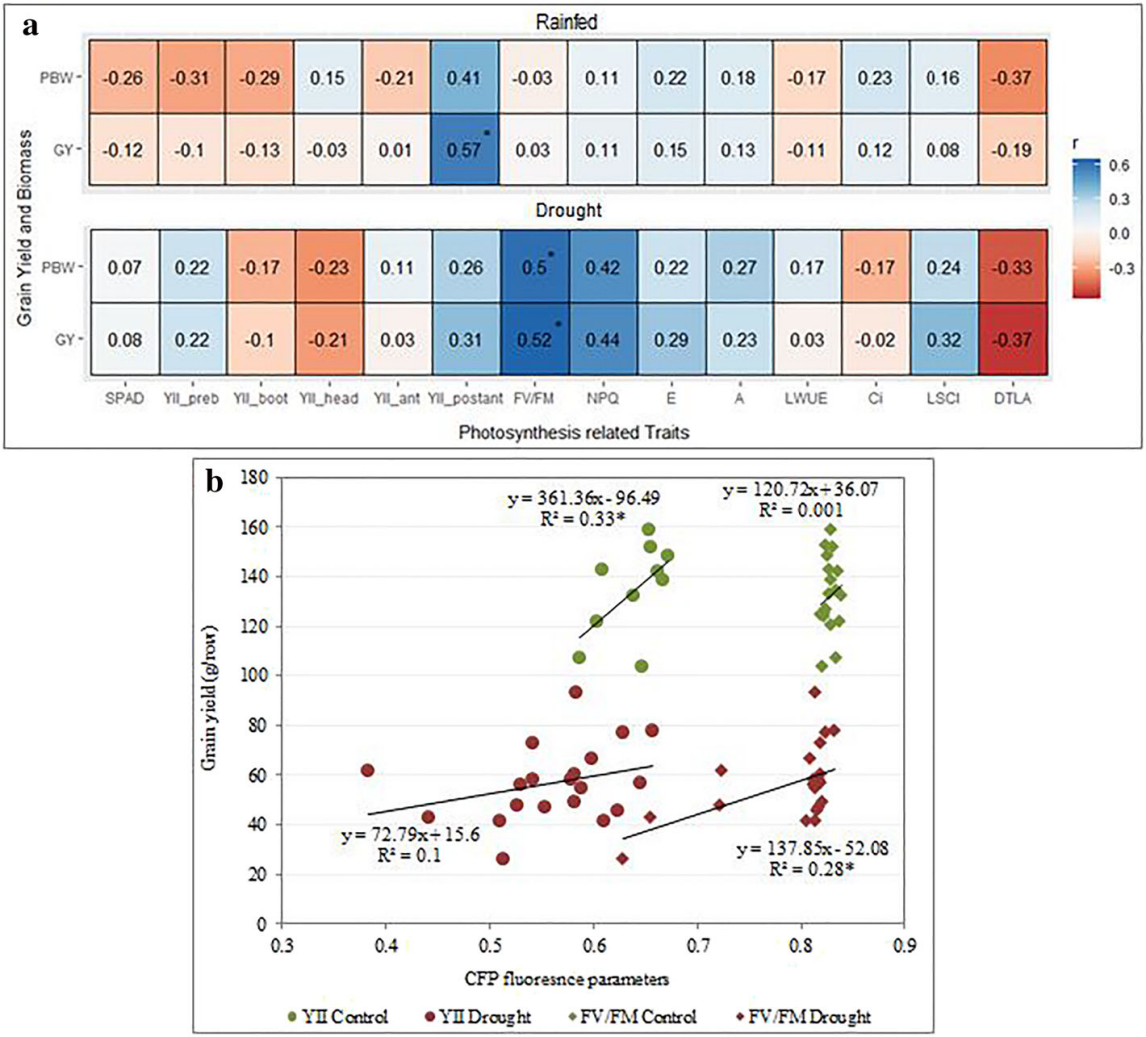
(a) Pearson correlation coefficient of photosynthetic traits vs aboveground yield (grain yield and plant biomass weight) under rainfed conditions (up panel) and drought conditions (lower panel). The legend on the right indicates the correlation coefficients. (b) Relationship between GY versus YII and F_V_/F_M._ The abbreviations of traits name are listed in Tables S2 and S3. The correlation coefficients significance level (*p* < .05) is indicated by *.

To evaluate the relationship between photosynthetic traits with PBW and GY, the correlation coefficients based on cultivars means were calculated (Figure 3a). GY and YII were found to be significantly and positively correlated at post‐anthesis. For instance, correlations between YII measured at post‐anthesis stage and GY were stronger than the ones of YII measured at earlier stages and GY under both control and drought conditions. Under drought conditions, significant correlations were detected between F_V_/F_M_ with GY and PBW at anthesis. Although not significant, NPQ had higher correlation with GY under drought than under control conditions (Figure 3a). Further regression analysis between GY versus YII and F_V_/F_M_ showed that YII significantly explained the variation in GY under rainfed, while under drought, the change in GY was rather explained by F_V_/F_M_. The dispersions of scatter points across the regression lines indicated higher genetic variation for both traits under drought than rainfed conditions.

### 3.4 Breeding has contributed to improve photosynthesis and drought stress tolerance in wheat

**FIGURE 4:**
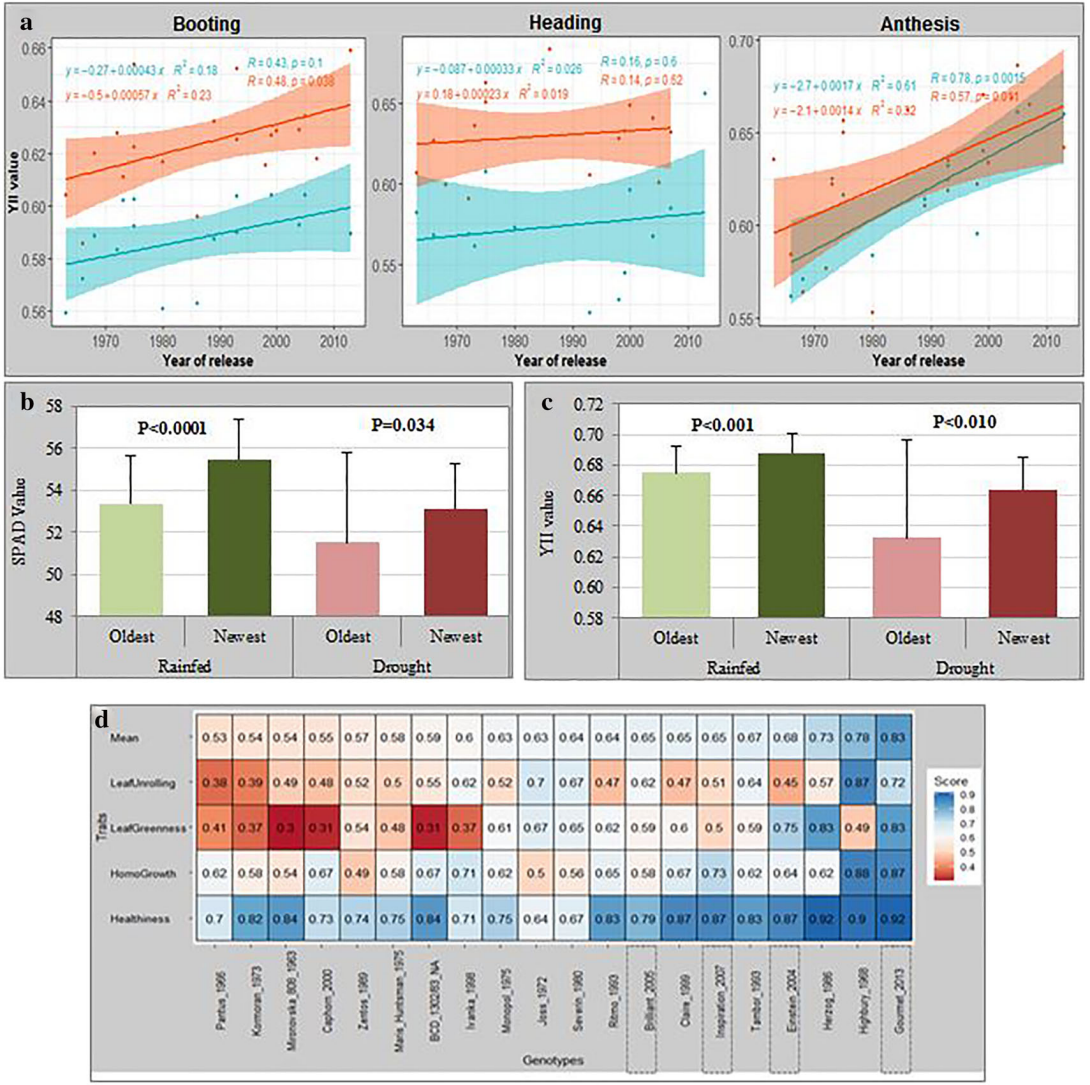
(a) Regression plots showing temporal trends in effective quantum yield of PSII among 20 winter wheat cultivars in relation to the year of cultivar registration under two contrasted water regimes. The slopes of the linear regression lines (orange line for drought and green line for rainfed) are referred to as absolute breeding progress. Boxplots of oldest versus newest released cultivars under rainfed and drought conditions for (b) YII and (C) SPAD values screened at heading/anthesis for the whole population; (D) heatmap representation of the average of visual scores of developmental traits screened over both experimental years showing a gradient of cultivars with lower relative scores in red (sensitive cultivars) to high relative scores in blue (tolerant cultivars). The error bars in panels (b) and (c) represent the standard deviations from the mean. The Mean in panel (d) represents the average of the scores of the four developmental traits.

We investigated the contribution of breeding to photosynthesis and DT in wheat by comparing slopes of the linear regression between the year of release and the cultivars mean value of the traits of interest. The results indicate that BP from 1963 to 2014 correlates positively with the effective quantum yield of PSII. The newer released cultivars exhibiting higher photosynthetic activity potential than the older cultivars across all GS (Figure 4a). The highest slopes were detected at anthesis under drought conditions (Figure 4a). The years of release significantly explained 32% of the variations (*R* ^2^) for YII at anthesis and 23% at booting under drought stress. Under rainfed conditions, year of release explained 62% and 18% of the variation for YII at anthesis and booting, respectively. Moreover, the modern (newest) cultivars had significantly higher YII and chlorophyll content than older ones when their values were compared. Interestingly, for YII, the difference between the newest and oldest cultivars groups is higher under drought than rainfed conditions (Figure 4b,c). In addition, recent released cultivars also recorded higher leaf greenness, healthiness, and leaf unrolling traits scores than the old released cultivars, suggesting their higher resilience to drought over the older cultivars (Figure 4d).

### 3.5 GWAS uncover QTL of photosynthetic trait linked to DT on chromosome 3A

The analysis of the genetic data revealed the sub‐genome B had the highest number of SNP markers (11,887) with chromosome 5B being the largest (2,131), whereas Genome D (2,364), especially 4D (104) had the lowest number of SNPs (Figure S4). The LD decay that determines the resolution of association mapping was estimated at 19, 38, and 17.5 megabase pairs (Mbp) to background level of *r* ^2^ = .1, across the A, D, and B genome, respectively (Table Sxl1; Figure S5).

Relative kinship within the diversity panel was evaluated based on pairwise kinship between cultivars calculated with 24,216 SNP marker set. From 19,900 pairwise kinships calculated among cultivars of the panel, 61.20% of the total number of kinship estimates were below 0, and 38.35% were higher than 0 and less than 1 (Figure S6A). The decline in the frequency of higher pairwise kinship coefficient was continuous till 1, and few estimates were higher than 1, suggesting a weak genetic relationship among the cultivars of the panel (Figure S6B). Population structure inferred using the STRUCTURE algorithm and Evano test (ΔK) methods indicated two sub‐populations within the 200 wheat cultivars (Figure S7AB), with the first and second PCs explaining 11.09 and 4.15% of the genetic variance, respectively. With membership coefficient allotments of *Q* > .8, 99 and 25 cultivars were inferred to belonging to sub‐population 1 and 2, respectively, and 76 cultivars with *Q* < .8 were designated as admixture. The two distinct defined sub‐groups related to the origins of the cultivars with sub‐group 1 comprising entries originating from Europe (F*st* = .3133), while sub‐group 2 included entries outside Europe (F*st* = .0745). The cultivars were color coded according to this structure result and plotted with PC1 versus PC2 (Figure S7C). The Q1 values of ancestry coefficient (*Q* matrix) given by population structure analysis at *K* = 2 were color coded and mapped with the geographic origins of cultivars (Figure S7D).

**FIGURE 5:**
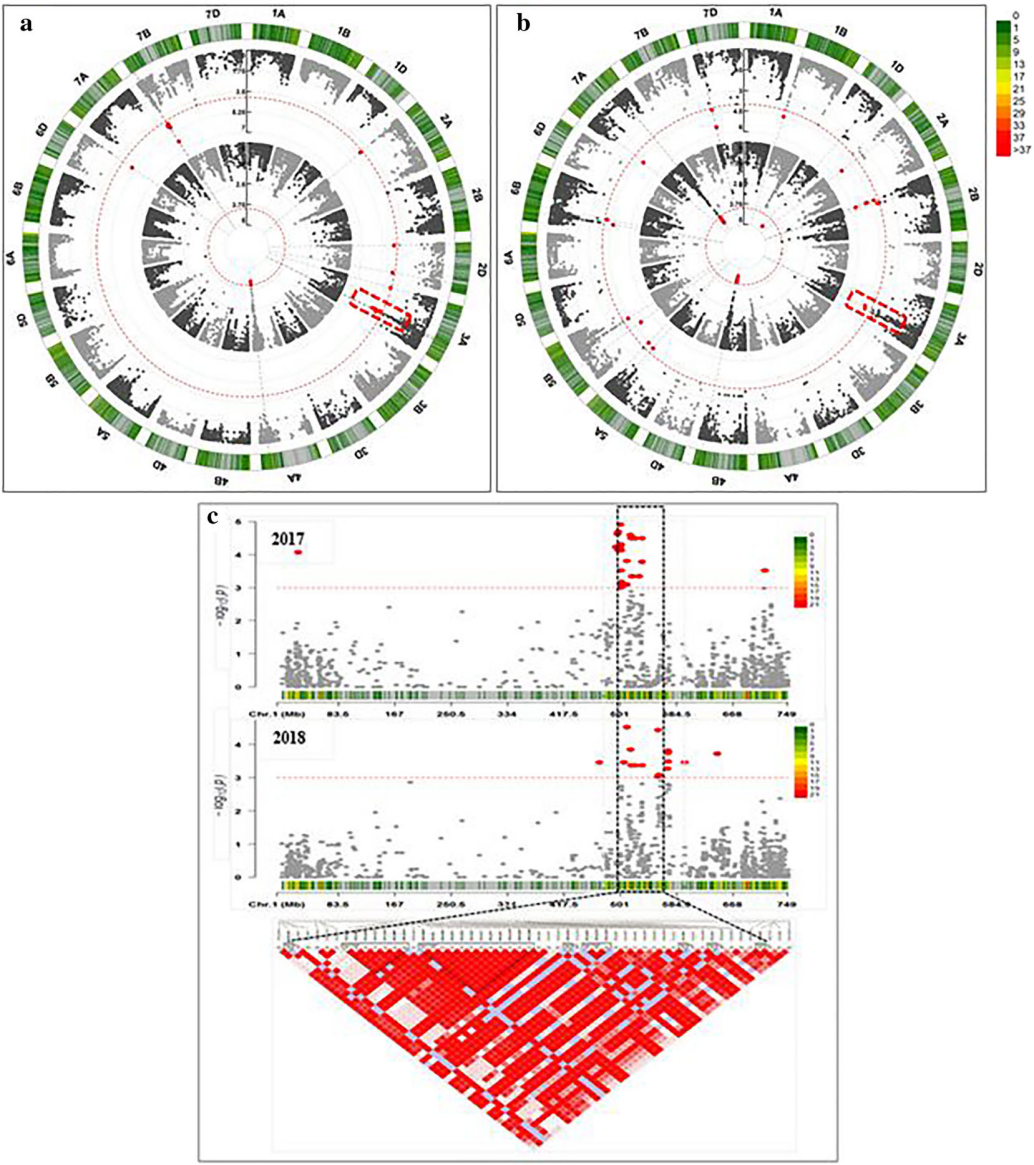
Circle Manhattan plot for YII under control (inside track) and drought (outside track) including significant marker‐trait associations (MTAs) in (a) 2017 and (b) 2018 showing drought inducible quantitative trait locus (QTL) on chromosome 3A. (c) Manhattan plot showing a hotspot of associated SNPs on 3A region of 22 Mbp length delimited from AX‐158597824 (510.691 Mbp) to wsnp_Ex_rep_c66865_65263145 (533.624 Mbp) associated with YII under drought stress in 2017 and 2018.

GWAS were conducted to identify QTL that are significantly (*p* < 10^−4^) associated with the response of the photosynthetic traits to drought stress (Table Sxl2). A total of 51 and 117 MTAs, representing 11 and 23 QTL regions, respectively, were significantly associated with the photosynthetic traits measured at anthesis growth stage under control and drought conditions, respectively. All the detected 117 MTAs were induced by drought effect as they were not present under control conditions. The highest number of significant MTAs was obtained for YII under both water regimes (Table Sxl2). The drought inducible QTL interval ranging from 510.691 to 533.624 Mbp on chromosome 3A was associated with YII traits (Figure 5; Table Sxl2). The QTL regions on chromosome 7A covering genetic interval between 267.570 and 286.152 Mbp were associated with chlorophyll content under control conditions (Figure S8), whereas another one spanning on 10.480 Mbp length from 583.204 to 593.684 Mbp was detected for YII (Table Sxl2).

### 3.6 Effects of genotype by treatment and epistatic interactions to photosynthetic traits

GWAS was run to detect QTL involved in marker by treatment interactions effect. A total of 11 genomic regions comprising 128 significant markers (FDR < 10^−4^) harbor QTL for chlorophyll content and YII that interact with water‐treatment (Table Sxl3). The highest number (100) of marker * treatment interactions was detected on chromosome 3A for chlorophyll content and YII traits (Table Sxl3 and 4). The marker * treatment interaction effect analysis on the associated chromosome 3A LD‐block between the interval (515.889 and 516.803 Mbp) with the SNP peak AX‐158576783 indicated that genotypes with major alleles (TT) recorded significantly higher values than those with minor alleles (CC) under drought conditions, whereas the contrary pattern was observed under rainfed conditions (Figure S9).

**FIGURE 6:**
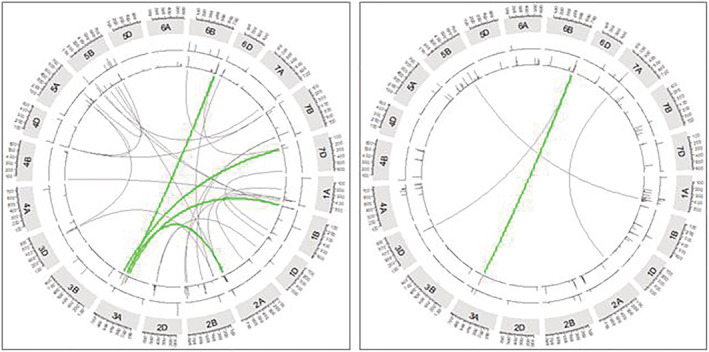
Circular plot showing the epistatic interactions SNPs with the corresponding positions on the genetic map of wheat. Wheat chromosomes 1A–7D are shown in a clockwise direction in the Circos diagram. Green‐colored connections represent epistatic loci on chromosome 3A controlling YII (left panel) and SPAD values (right panel). Gray‐colored connections represent epistatic interactions on other chromosomes. The first track line after the chromosome name track is showing the significant (10^−4^) epistatic loci detected under rainfed while the following track is showing the ones detected under drought conditions.

Drought has triggered a total of 19 epistatic interactions between SNP loci at 25 QTL regions for both YII and chlorophyll content (Figure 6; Table Sxl8). Specifically, 26 SNPs located on 12 chromosomes were involved in 15 epistatic interactions for effective quantum yield of PSII, whereas 4 epistatic interactions included 7 SNPs on 6 chromosomes for chlorophyll content. Among SNPs with epistatic interactions were 4 SNPs that as well detected for the main‐effects in the GWAS analysis performed for YII (Table Sxl8). SNP locus AX‐111134276 located at 556.662 Mbp on 3A, which had significant effect on YII via GWAS under drought conditions, exerted high epistatic interactions with wsnp_Ku_c28854_38769308 at 690.958 Mbp on 6B. Likewise, the locus AX‐111134276 interacted epistatically with AX‐158588791 at 695.492 Mbp on 6B for chlorophyll content. Both wsnp_Ku_c28854_38769308 and AX‐158588791 are located in the same QTL region.

Analysis of the effect of the interacting SNP pairs (wsnp_Ku_c28854_38769308 [G/A] at 690.958 Mbp on 6B and AX‐111134276 [G/A] at 556.662 Mbp on 3A) on YII indicated that combination A * G (minor allele * major allele) increased the YII value with 17.72% higher than the combination G * A (major allele * minor allele), which decreased it. In the same order, the combination of G * G (minor allele * major allele) of SNP pairs (AX‐158588791 [T/G] at 695.492 Mbp on 6B and AX‐111134276 [G/A] at 556.662 Mbp on 3A) increased the chlorophyll content by 14.60% compared with T * A combination (Table Sxl8). In addition, AX‐109950638 (G/A) interacted epistatically with AX‐109950638 (G/T) at 699.434 Mbp on 2A for YII. Genotypes with alleles pairs G * G (major allele * major allele) had higher YII than the one with A * G and A * T (Table Sxl8).

### 3.7 Breeding has improved genetic factors involved in photosynthesis and DT

**FIGURE 7:**
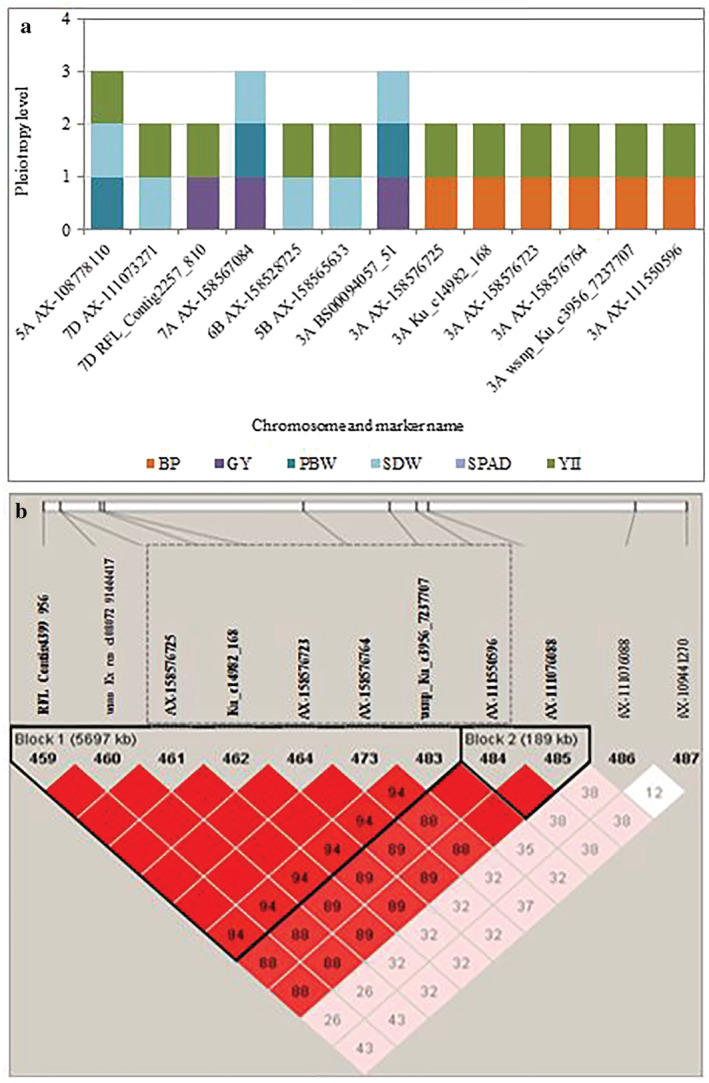
(a) Representation of most pleiotropic SNPs detected under drought, among which some are involved in breeding progress. (b) Chromosomic region of 5.847 Mbp length on 3A from RFL_Contig4399_956 (496.705 Mbp) to AX‐111076088 (503.027 Mbp) harbored six SNPs (gray square) associated with breeding progress and YII. Two haplotypes blocs were found in this region, pairwise D′ between SNPs of LD block are displayed.

**FIGURE 8:**
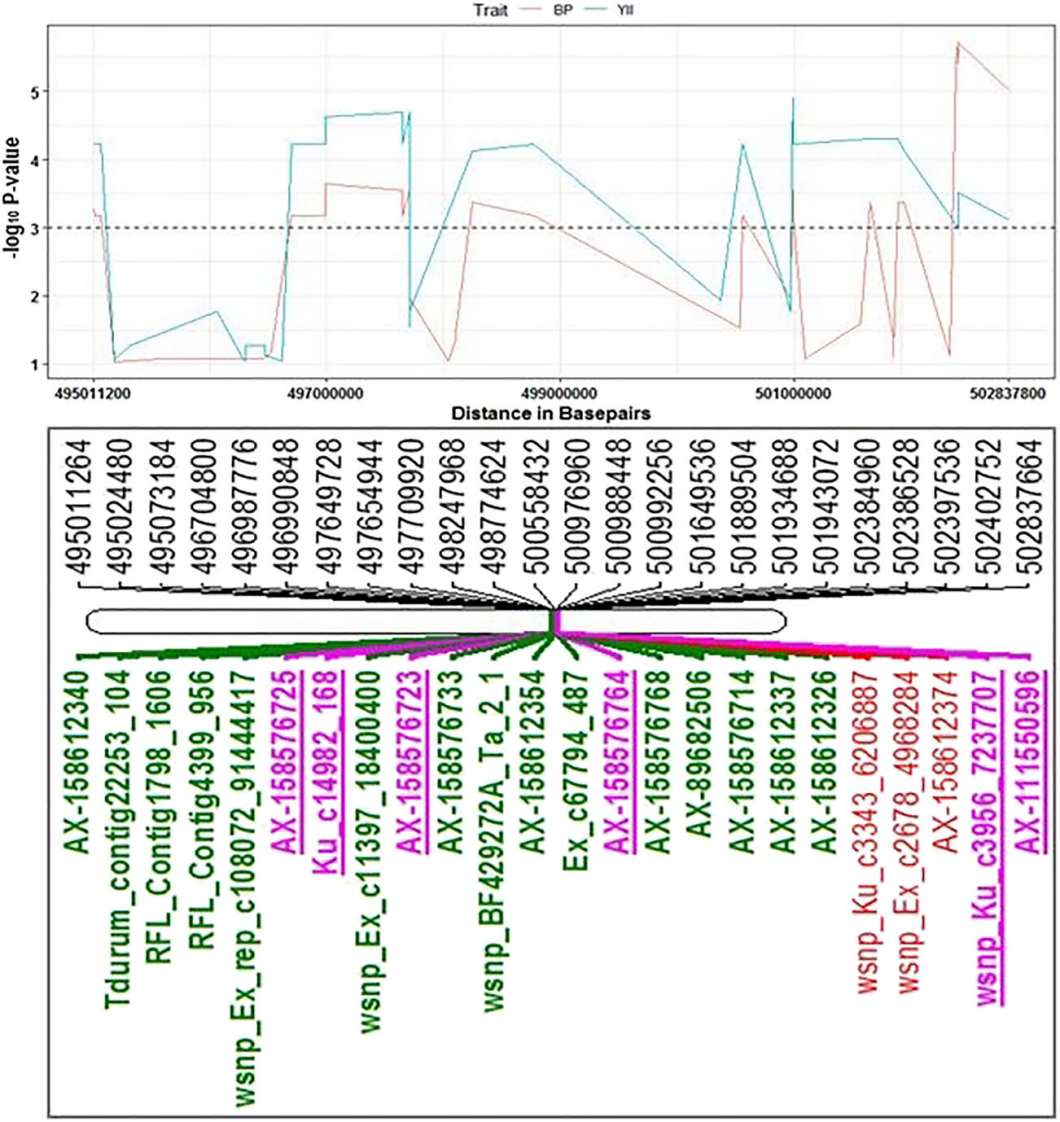
Illustration of genomic region on chromosomes 3A harboring pleiotropic SNP for breeding progress (BP) and YII. The upper panel presents genome‐wide association studies (GWAS) –log_10_ *p* values of significant SNPs between 495 and 503 Mbp associated with BP (red color) and YII (green color). The dotted line (−log_10_ *p* value = 3) indicated the threshold considered to find significant marker‐trait associations (MTAs) with pleiotropic effect for BP and YII. The lower panel is the map positions of drought inducible SNPs associated with evaluated traits. Map distance (in base pairs) is shown on the upper part of the chromosome bar, and the SNP names are under the chromosome bar. “Underlined and bold” SNPs names are pleiotropic for YII and BP; the color of the SNPs names indicated the category of traits the SNP is associated with [“red” = BP; “green” = YII; “underlined purple” = BP + YII].

**FIGURE 9:**
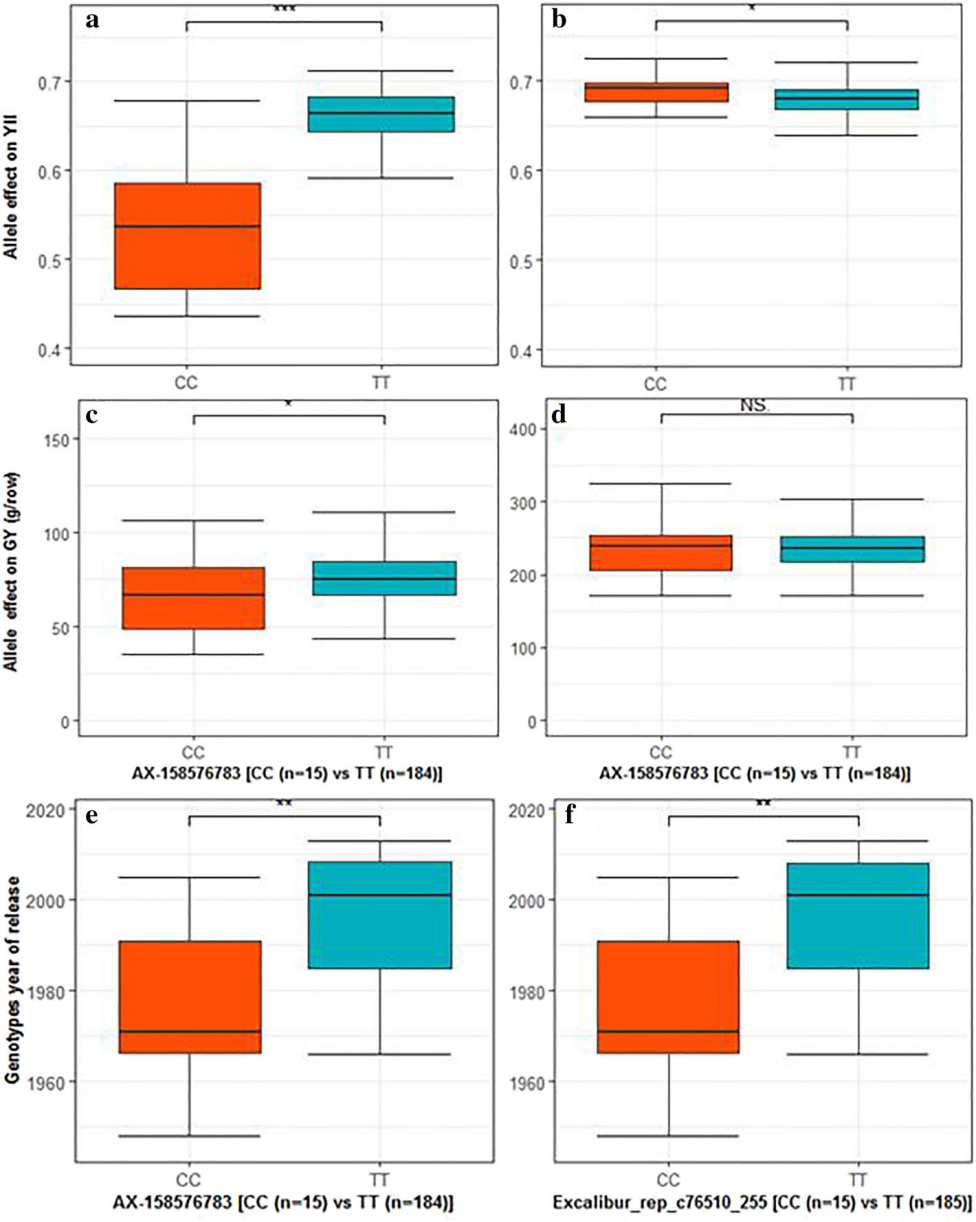
Allelic effect of the chromosome 3A SNP AX‐158576783 (515.889–516.804 Mbp) on YII under drought (a) and rainfed (b); and its effect on GY under drought (c) and rainfed (d); (e,f) The SNP AX‐158576783 alleles distribution by cultivars year of release in the wheat panel. Two‐sample *t‐*test *p* values show significant difference (* for *p* < .05; ** for *p* < .01; and *** for *p* < .001) between major (TT) and minor (CC) alleles.

Stable QTL that was detected in 2017 and 2018 trials and showing pleiotropic effect on several traits including SDW, PBW, and GY was identified and further analyzed. A total of 57 and 15 SNPs located on chromosome 3A and 7A, respectively, were stable QTL and/or exhibited pleiotropic effects on the traits (Table Sxl5). A QTL region spanning 5.847 Mbp from RFL_Contig4399_956 (496.705 Mbp) to AX‐111076088 (503.027 Mbp) on chromosome 3A had a pleiotropic effect on BP and YII (Figures 7 and 8). The LD analysis performed showed that the associated genomic region on chromosome 3A that showed pleiotropic effect on YII and BP is in high LD (*r* ^2^ > .86). Comparison of the allelic effects on traits of the SNPs in 3A haplotype‐block region (peak marker AX‐158576783 at 515.889 Mbp) revealed that genotypes with TT (major) alleles significantly contributed to higher YII and GY when compared with genotypes with the CC (minor) alleles. The observed allele effect was found to be stronger under drought conditions (Figures 9a,c and S10A,C) than under control conditions (Figures 9b,d and S10B,C). The mean GY and YII of genotypes with TT alleles are 76.68 g/row and 0.66, respectively, whereas the genotypes with CC alleles had 65.92 g/row and 0.53 for GY and YII. Further analysis showed that the favorable (TT) alleles were prominently present in the newer released cultivars, whereas the unfavorable alleles (CC) were present in old cultivars (Figure 9e,f). When evaluating the BP realized between 1963 and 2013 (oldest and newest year of release of the core set, respectively) in relation with the allele effects (TT vs. CC), an increase of 16.33% (GY) and 23.11% (YII) in crop productivity was estimated.

**FIGURE 10:**
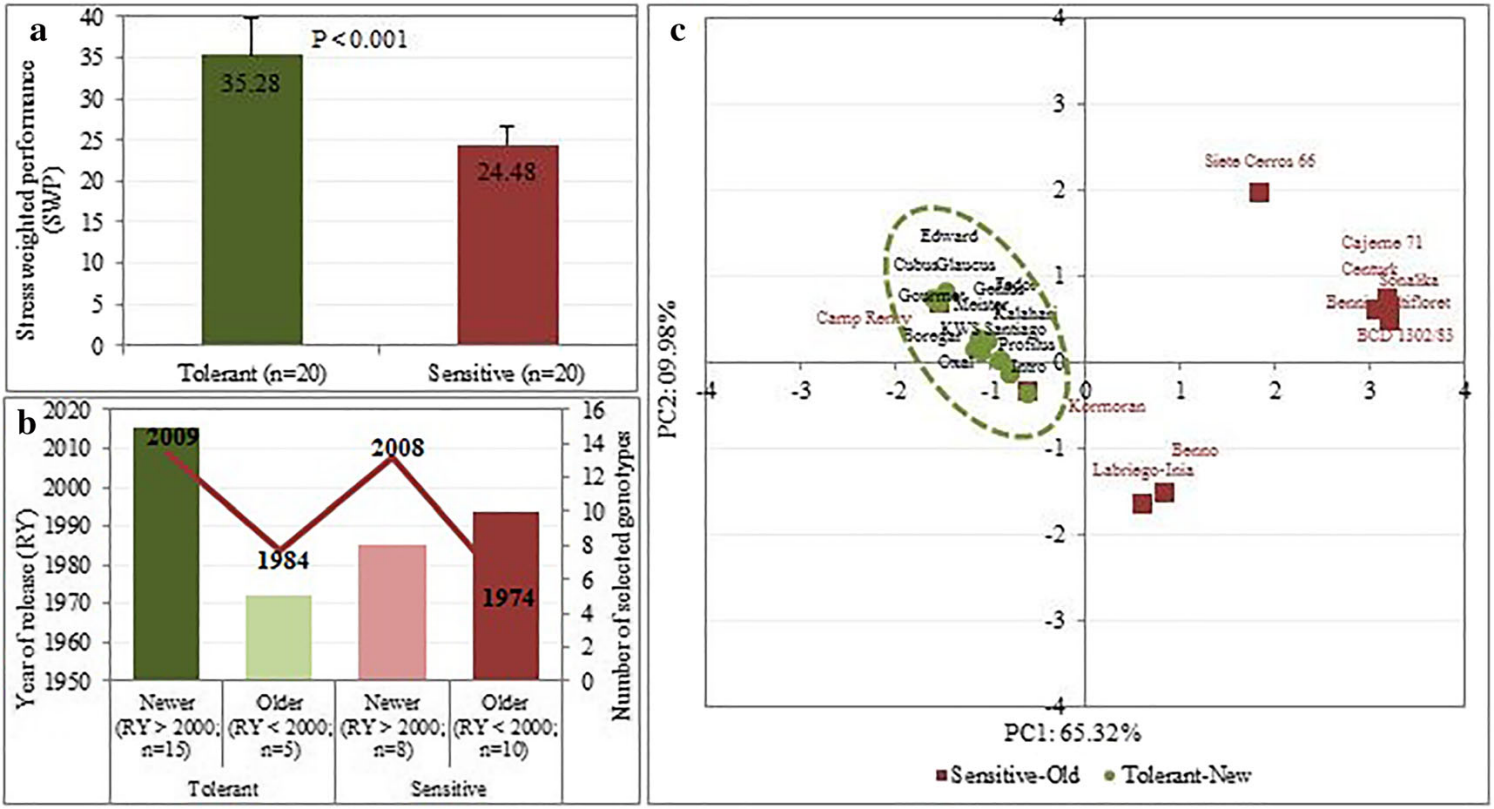
(a) Representation of 20 drought‐tolerant (dark‐green) and 20 drought‐sensitive (dark‐red) cultivars groups based on their stress weighted performance (SWP) estimates; the *p* value indicates significant difference between both groups. (b) Bar plot of newer (dark‐green) and older (dark‐read) released cultivars based on SWP estimates. The dark‐green color showed that new released cultivars are prominent among the drought‐tolerant cultivars, whereas old ones in dark‐red color are mostly present in the drought‐sensitive group. The light‐green color showed some old‐released cultivars that are drought‐tolerant, whereas the light‐red indicated some new‐released that are drought‐sensitive. (c) Biplot of principal component analysis based on 28 marker‐trait associations (MTAs) of breeding progress (BP) separated 13 drought‐tolerant and new released (in green circle) and 10 drought‐sensitive and old cultivars (dark‐red square).

The DT status of the cultivars in the studied panel was calculated for GY, SDW, PDW, chlorophyll content, and YII using the SWP index, and cultivars were ranked as tolerant (highest SWP) to sensitive (lowest SWP). Based on the SWP index, 20 cultivars with SWP index > 31.99 were considered tolerant, whereas another 20 with SWP index < 27.03 were identified as sensitive genotypes (Figure 10a). Interestingly, most selected drought‐tolerant cultivars were recently released, whereas the old release cultivars were mostly observed in the sensitive group (Figure 10b). PCA constructed with the selected tolerant and sensitive cultivars using the SNP markers associated with BP trait separated the cultivars into two groups. The first two components explaining 75.01% of the total variation and the grouping were based on the DT status of the cultivars. The recently released cultivars being drought‐tolerant (in green/circle‐shaped) were mostly clustered in the left side of the plot, whereas the old released cultivars and most sensitive were distributed at the right side of the plot (Figure 10c).

### 3.8 Candidate genes in the chromosomic regions harboring stable and pleiotropic MTAs

The candidate genes in the region harboring stable and pleiotropic MTAs were retrieved, and the result is presented in Table Sxl6. A total of 225 HC genes including 58 on chromosomes 3A were retrieved from associated QTL regions. The associated region for YII spanning 12.912 Mbp on chromosome 3A contains 42 genes involved in response to oxidative stress. The 3A AX‐158576783 haplotype‐block (515.889–516.804 Mbp) contains genes whose gene ontology (GO) terms are related to cellulose synthase, electron transport, coupled proton transport mitochondrial respiratory chain complex I assembly, contain WRKY transcription factors, and chaperone protein dnaJ, which protect proteins from external stress. Chromosomic region of the stable SNP peak Excalibur_rep_c68899_1400 on chromosome 2B underlying GY and YII harbored 31 genes (Table Sxl3) mainly involve in peroxidase and non‐specific serine/threonine protein kinase kinase activity, and stress protector genes such as heat shock 70 kDa protein. SNP peak RFL_Contig2257_810 on chromosome 7D, although not in LD block with other significant candidate loci, has a pleiotropic effect for YII and GY under drought. This SNP co‐segregates with 13 HC genes majorly involved in carbonic anhydrase and oxidation‐reduction process heme binding, carbonate dehydratase activity, ATP binding protein, protein phosphorylation, and recognition of pollen.

The in silico analysis of the 3A and 5B chromosomic regions with high interaction marker * treatment effect revealed high sequence homologies to genes involved in drought stress response and photosynthetic activity (Table Sxl7). A total of 536 HC genes were found in the five regions where SNP peak for interaction effect was located. Thirty‐two of these genes are involved in plant response to environmental stress and defense mechanisms including heat shock protein and transcription factors, zinc finger C3H1 domain, and disease resistance protein. We found genes category involved in phosphorylation, glycerol metabolic process, and electron transport, whose actions play important role in photosynthetic activity.

BLAST searches indicate that most of significant epistatic loci are located in the vicinity of genes involved in photosynthetic activity, particularly in oxidation–reduction process and phosphogluconate dehydrogenase (decarboxylating). These QTL regions harbor ferredoxin reductase‐type FAD‐binding domain and alternative NADH‐ubiquinone oxidoreductase, which catalyzes the oxidation of mitochondrial NADH. In addition, universal stress proteins, disease resistance protein, and nucleotide‐diphospho‐sugar transferases were found (Table Sxl9).

## 4 DISCUSSION

Water stress is a major threat to wheat production that threatens the global food security. The use of diverse wheat panel is important to assess the genetics of wheat's response to drought stress and to uncover the mechanistic basis that contributes to DT in wheat. Considerable effort has been made to quantify drought effect on yield lost, but few studies have focused on unveiling the genetic factors that have contributed to the BP achieved for DT between 1950 and 2013. In this study, we investigated the genetic variation in the photosynthetic activity of wheat under drought stress, to identify QTL with pleiotropic and stable effect on the photosynthesis and yield and shed more light on the contribution of breeding to DT.

### 4.1 Phenotypic variation in response to drought stress

Drought had significant effect on chlorophyll content and effective quantum yield of PSII across GS with the observed effect of 3.47% and 27.03%, respectively. Result showed that the photosynthetic rate decreased by 29.53% due to drought which resulted in 79.65% GY reduction as reported by Koua et al. (2021). Previous studies have shown the direct and positive link between photosynthesis ability and yields in crop under drought stress (Barnabás et al., 2008; Farooq et al., 2014). The reduction of photosynthetic parameters under prolonged drought conditions is expected at several levels as drought affects all biological processes in chloroplast including disorder of the electron transfers in PSII (Balla et al., 2014; Yang et al., 2007). Reduction of photosynthetic activity under drought stress is caused by accelerated leaf senescence due to the breakdown of chlorophyll molecules, affecting the stay green state of the plant, particularly the flag leaf (Yang et al., 2001). Excessive drought may increase leaf temperature, which was the case in our study. Harding et al. (1990) have shown that high temperature caused accelerated aging, leading to the activation of proteolytic enzymes, protein degradation, and chlorophyll losses. The reduction of chlorophyll content leads to a decrease in the chlorophyll fluorescence level, which, in turn, lessened the effective quantum yield of PSII and prevented the reduction of NADP^+^ to NADPH and the formation of ATP (Pinto et al., 2020). Another reason for the reduction of photosynthesis under stress conditions is the stomatal closure to prevent water loss, leading to a lower internal CO_2_/O_2_, hence making carbon assimilation less efficient during the Calvin Cycle reaction of photosynthesis (Araus et al., 2008).

Under drought stress conditions, the correlations among photosynthetic related traits were higher than those under rainfed conditions, suggesting that wheat has different genetic and mechanistic response to both environments. Identification of these mechanisms will support DT breeding in wheat. Specially, chlorophyll content exhibited higher correlations with fluorescence parameters under drought conditions, suggesting that the high throughput measurements of this trait can serve as a good surrogate for determining the chlorophyll content. The CV among the genotypes ranged from 5.95% to 21.88% and 6.20% to 34.75% under control and drought conditions, respectively. The recorded heritability estimates of the traits ranged from moderate (6.37%) to high (92.57%), suggesting that the variational response to drought can be genotypically exploited. The marker‐based heritability, which is relevant in dissection of complex traits (Kruijer et al., 2015), revealed that a larger portion of phenotypic variance is ascribed to genotypic variance for chlorophyll content under both water regimes. These results highlight the existence of high variability in cultivar's responses to drought stress that can be exploited through GWAS to develop drought‐tolerant cultivars.

The presented investigations revealed plant photosynthetic traits such as YII, F_V_/F_M_, and NPQ at the post‐anthesis were positively strongly correlated with the PBW and GY. At anthesis and post‐anthesis, higher photosynthetic activity in plants is relevant for increased GY as it affects the key yield components (Lichthardt et al., 2020). Similar relationships between photosynthetic related traits, namely, chlorophyll content, leaf CO_2_ assimilation (A), and YII and GY, were reported (Méndez‐Espinoza et al., 2019). Large proportion of assimilates necessary for filling the grain are provided by photosynthesis in the leaves and by the translocation of reserves stored during the pre‐ and/or post‐anthesis periods (Maydup et al., 2012; Tambussi et al., 2007; Zhang et al., 2006).

The photosynthetic efficiency increased simultaneously with the BP, indicating that wheat breeding has contributed to the selection of wheat cultivars that have favorable variations of alleles that not only contribute to improve GY but also enhanced photosynthetic ability. For instance, the modern cultivars (released after 2010) that exhibited higher GY potential on our previous study (Koua et al., 2021) also have higher chlorophyll content and effective quantum yield of PSII values than the old cultivated (released before 1980) in the present study (Figure 4). Studies have reported high yield performance of modern over older cultivars owing to their higher photosynthetic capacity during the milk‐grain stage to maturity (Araus et al., 2008; Sanchez‐Garcia et al., 2015). Comparison of chlorophyll content and YII values of cultivars released before 1980 and the ones released after 2010 confirmed that modern cultivars have significantly higher values in both parameters. Interestingly, for YII, the difference between these two contrasting groups was higher under drought than under control, suggesting breeding has increased wheat GY under drought conditions by accumulating genetic variants that have favorable effects on photosynthetic activity under less optimal conditions (Voss‐Fels et al., 2019).

### 4.2 Population structure and LD pattern

Two main sub‐populations were identified in the studied panel. The groupings were based on the geographic origin of cultivars including Europe and outside Europe clusters and an admixture group between both sub‐populations. The *F*_*ST*_ value among cultivars originating from Europe was found to be higher (.31) than those emanating from outside Europe (.07), indicating of high genetic diversity within the European materials due to germplasm exchange between breeding programs and limited selection pressure and genetic drift (Chao et al., 2017; UPOV, 1991). The low intra‐population *F*_*ST*_ values suggested a week population structure in the evaluated panel, which suggests that individuals within each sub‐populations share some alleles that may have been undergoing continuous selection in wheat breeding history. Moreover, the LD of the studied panel decayed after 19.0, 38, and 17.5 Mbp for A, B, and D genomes, respectively, revealing that the LD decay of genome B was slower than A and D. Similar trends in the genomes LD decay were found in earlier studies performed the same germplasm genotyped with 15K chip SNP marker set (Voss‐Fels et al., 2019). Inclusion of principal components as population structure matrix and a kinship matrix in the GWAS mixed model would minimize the occurrence of spurious or false‐positive associations (Gajardo et al., 2015).

### 4.3 Genetic variant with improved photosynthetic activity has conferred DT

GWAS of chlorophyll content and fluorescence parameters identified 51 MTAs and 117 MTAs corresponding to 11 and 23 QTL regions under rainfed and drought conditions, respectively, with the highest number of MTAs on chromosome 3A. Most of the detected MTAs were only present under drought stress conditions, suggesting that they might be drought inducible QTL. The genetic control of chlorophyll fluorescence parameters could vary considerably between drought‐stressed and non‐stressed plants (Czyczyło‐Mysza et al., 2011). Interestingly, chromosome 3A harbored QTL associated with all the evaluated chlorophyll fluorescence parameters under drought conditions in the present study and was also found to be associated with Fmax and YII by Czyczyło‐Mysza et al. (2011). Moreover, the associated QTL regions on chromosomes 1B, 7A, 7B, and 7D (Table Sxl4) were also proximal to QTL that were previously reported for chlorophyll content, water use efficiency, osmotic adjustment, and chemical desiccation tolerance in several studies (Hao et al., 2003). Similar to previous reports (Czyczyło‐Mysza et al., 2011; Hao et al., 2003), chromosome 7A (263.733 to 285.609 Mbp) harbored QTL hotspot constantly associated with chlorophyll content, YII under rainfed conditions. Contrary to the present research, most of these QTL mappings were done in biparental mapping populations consisting of double haploids (Christopher et al., 2016; Czyczyło‐Mysza et al., 2011).

The associated region on chromosome 3A spanning from 510.691 Mbp (AX‐158597824) to 533.624 Mbp (wsnp_Ex_rep_c66865_65263145) seems to be novel as it has not been reported in earlier studies. This region harbor genes involved in electron transport, and contain WRKY transcription factors, and chaperone protein dnaJ that play a role in protection against external stress. The WRKY transcription factors play significant role in molecular regulation of multiple stress responses in plants and have become promising candidate for crop improvement (Banerjee & Roychoudhury, 2015; Jiang et al., 2015; Phukan et al., 2016).WRKY clusters enhanced salt and DT in transgenic tobacco by regulating stomatal conductance and ROS levels (Chu et al., 2015; Li et al., 2015). Although chromosome 3A was not found in many reported studies on wheat, the consistency of the QTL hotspot in this region over 2 years in the present study supports it as a good candidate for higher photosynthetic activity under drought conditions.

The identified QTL in our study provides basis for further molecular breeding investigation as they co‐segregate with genes involved in plant response to abiotic stress such as production of stress‐related proteins under drought stress conditions and in oxidation‐reduction processes and carbohydrate metabolism‐related proteins (Cheuk et al., 2020). Specifically, on chromosome 7D, RFL_Contig2257_810 pleiotropic for Fmax, YII, and GY co‐segregated with carbonate dehydratase activity and β‐galactosidase activity. With reduced photosynthetic activity, it has been observed that the galactosidase activity could enhance sugars needed as energy source when photosynthates production is lower (Pandey et al., 2017).

## 5 CONCLUSION

The current study demonstrates important reduction of chlorophyll content, fluorescence parameters values, and photosynthetic efficiency under prolonged drought conditions. Genotypes responded differently to drought stress for the evaluated photosynthetic traits, which was confirmed with the visual scoring of developmental traits. The positive relationship between most photosynthetic traits such as chlorophyll content, YII, and F_V_/F_M_ screened at anthesis or post anthesis GS, and the PBW and/or GY support the importance of high photosynthesis in increasing biomass production not only under well‐watered field conditions but also under drought‐prone environments. Our results suggest the combination of both physiological and agronomic traits to efficiently select drought‐tolerant cultivars. Comparatively to most yield components and GY, breeding has significantly contributed to improve photosynthetic efficiency across all growth stages, but importantly at anthesis, under prolonged stress. GWAS unravel a hotspot of stable QTL on chromosome 3A involved in effective quantum yield of PSII, which is directly link to photosynthetic activity under drought conditions. Interestingly, several MTAs in LD block on 3A associated with breeding history showed pleiotropic effects with YII. Some of these MTAs had significant allelic effect on GY under drought conditions and co‐segregate with genes related to response to oxidative stress, cellulose synthase, aerobic respiration, and electron transport rate in the PSII chain. The loci and candidate genes identified in this research may facilitate the molecular breeding of drought‐tolerant wheat and improve wheat production under drought‐prone environments.

## CONFLICT OF INTEREST

The authors declare no conflict of interest associated with the work described in this manuscript.

## AUTHOR CONTRIBUTIONS

APK and MBS performed the data collection. APK analyzed the data and drafted the manuscript. SD provided the genetic map. AB, PK, SD, and JL designed the experiments. AB, APK, and JL interpreted the results. AB, BCO, SB, UR, and JL were responsible for the correction and critical revision of the manuscript. All authors read and approved the final manuscript.

## Supporting information


**Figure S1.** Genetic diversity of 200 winter wheat cultivars (in blue) including the core set of 20 genotypes (in red)

**Figure S2.** Pearson correlation coefficients between photosynthesis‐related under rainfed conditions (panel A). Correlation between photosynthesis‐related traits (gray square) and scored developmental traits under prolonged drought stress conditions (panel B).

**Figure S3.** Principal component analysis biplot using 11 photosynthesis and transpiration‐related variables under (A) rainfed, and (C) prolonged drought stress conditions. Cosines square of the variables contributing to the newly constructed principal components under rainfed (B) and (D) prolonged drought stress conditions.

**Figure S4.** SNP density across genomes of the studied winter wheat genotypes.

**Figure S5.** Sliding windows showing the rate of linkage disequilibrium decay among the 200 genotypes of the diversity set across A, B, D genomes.

**Figure S6.** (A) Classification of pairwise relative kinship into 3 classes; (B) Distribution of pairwise relative kinship estimates among 200 wheat cultivars.

**Figure S7.** Representation of the wheat panel population structure.

**Figure S8.** Illustration of marker‐trait association result for SPAD values.

**Figure S9.** Illustration of marker by treatment interactions on photosynthesis‐related traits

**Figure S10.** Allelic effect of Excalibur_rep_c76510_255 on YII.

**Data S1** Table Sxl1. Description of SNP number per chromosome and chromosomal LD.
Table Sxl2. Full description of detected significant MTAs (10–4) from GWAS under both water regimes in 2017, 2018 and with the means values of both years.
Table Sxl3. Summary of significant SNPs QTL detected in the marker*treatment interaction GWAS.
Table Sxl4. Table of QTL regions in the marker*treatment interaction GWAS and alleles combination effect.
Table Sxl5. GWAS results from four physiological traits and three final aboveground biomass traits highlighting SNPs with stable and pleiotropic effects.
Table Sxl6. Candidate genes in the QTLs regions with stable and pleiotropic effects.
Table Sxl7. Candidate genes in the QTLs with high significant marker*treatment interactions effects.
Table Sxl8. Table of significant SNPs in the genome wide SNP‐SNP epistatic interaction.
Table Sxl9. Candidate genes in the QTLs regions harboring SNPs with epistatic effects.

**Table S1.** List of measured leaf chlorophyll a fluorescence parameters.
**Table S2.** Complete description and abbreviation of evaluated traits in the study.
**Table S3.** Chlorophyll content and fluorescence ratio parameters across growth stages in 2017 and 2018 growing seasons.
**Table S4.** Dark and light adapted chlorophyll fluorescence ratio parameters measured at anthesis growth stage
**Table S5.** Stomatal conductance (gsw) dynamic across growth stage in 2018.
**Table S6.** Photosynthesis‐related parameters measured with the Licor 6800 at anthesis growth stage.

## Data Availability

The original contributions presented in the study are included in the article/Supplementary Materials. Further inquiries can be directed to the corresponding author/s. The following excel data are available at Zenodo Digital Repository: https://doi.org/10.5281/zenodo.5275829.
