# Supplementary figures and images for: Chromosome 3A harbors several pleiotropic and stable drought‐responsive alleles for photosynthetic efficiency selected through wheat breeding
Source: Plant Direct. 2022 Sep 2;6(9):e438. doi: 10.1002/pld3.438 (PMC9440346; doi:10.1002/pld3.438)

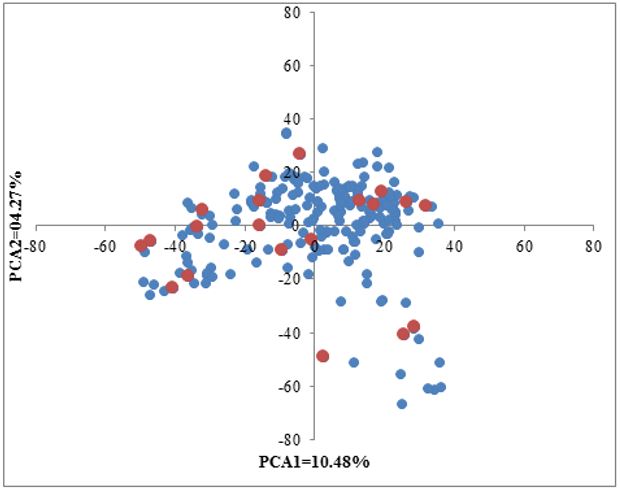

Supplement: Supplementary file 1 — Figure S1. Genetic diversity of 200 winter wheat cultivars (in blue) including the core set of 20 genotypes (in red) [file PLD3-6-e438-s010.jpg]

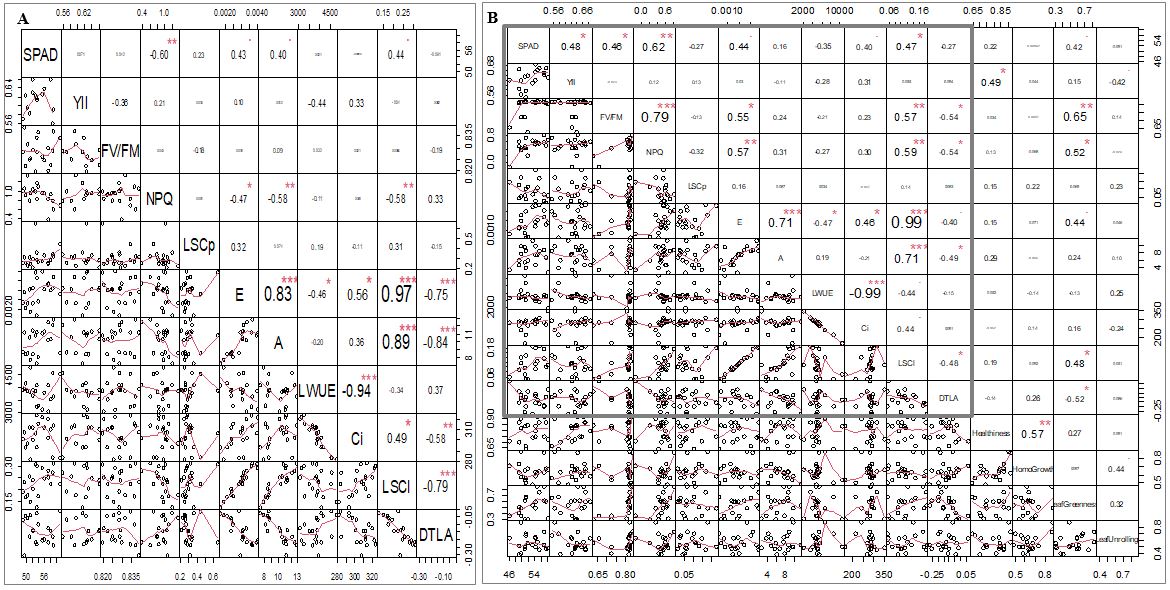

Supplement: Supplementary file 2 — Figure S2. Pearson correlation coefficients between photosynthesis‐related under rainfed conditions (panel A). Correlation between photosynthesis‐related traits (gray square) and scored developmental traits under prolonged drought stress conditions (panel B). [file PLD3-6-e438-s002.jpg]

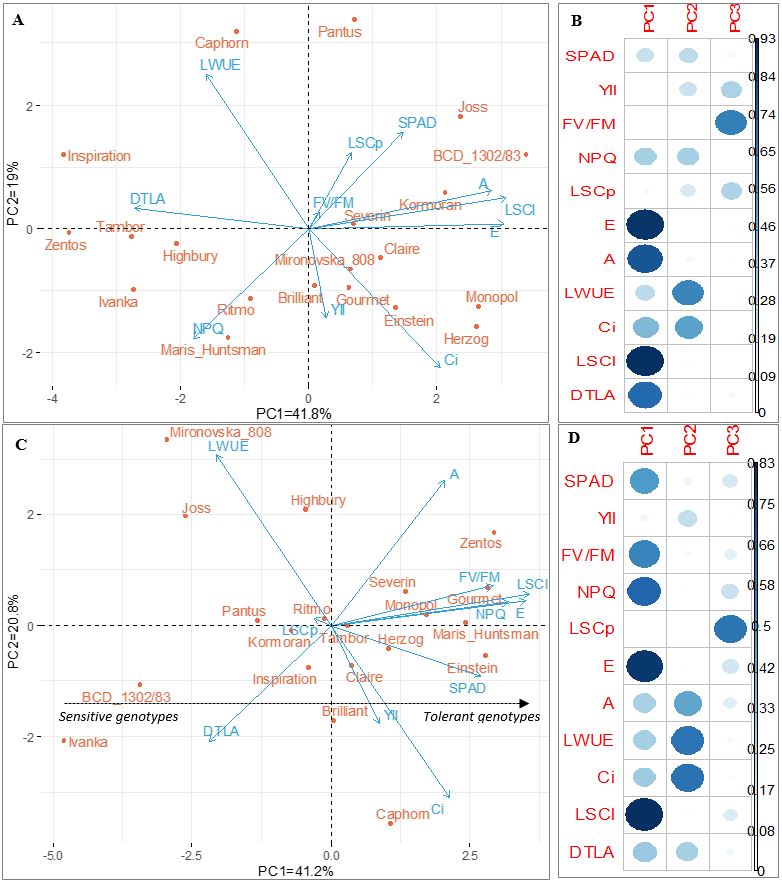

Supplement: Supplementary file 3 — Figure S3. Principal component analysis biplot using 11 photosynthesis and transpiration‐related variables under (A) rainfed, and (C) prolonged drought stress conditions. Cosines square of the variables contributing to the newly constructed principal components under rainfed (B) and (D) prolonged drought stress conditions. [file PLD3-6-e438-s007.jpg]

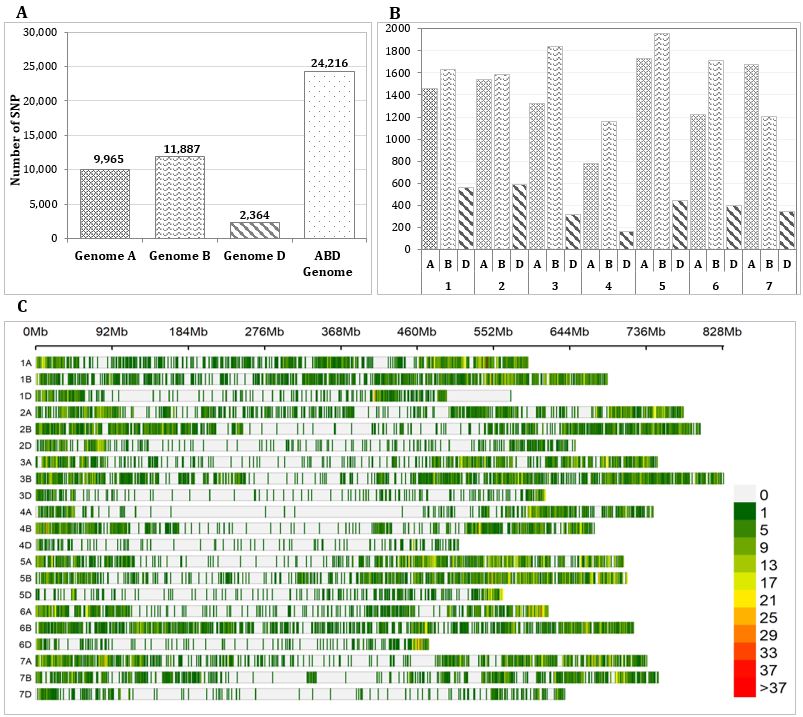

Supplement: Supplementary file 4 — Figure S4. SNP density across genomes of the studied winter wheat genotypes. [file PLD3-6-e438-s005.jpg]

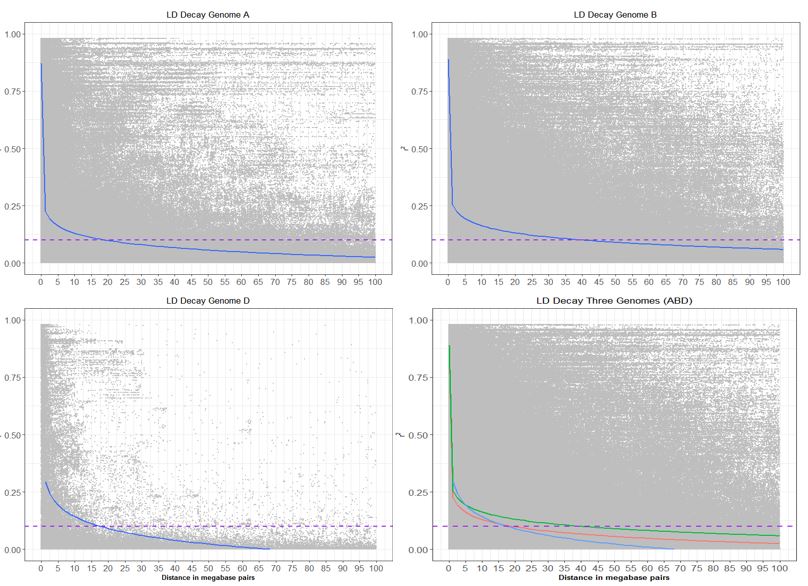

Supplement: Supplementary file 5 — Figure S5. Sliding windows showing the rate of linkage disequilibrium decay among the 200 genotypes of the diversity set across A, B, D genomes. [file PLD3-6-e438-s011.jpg]

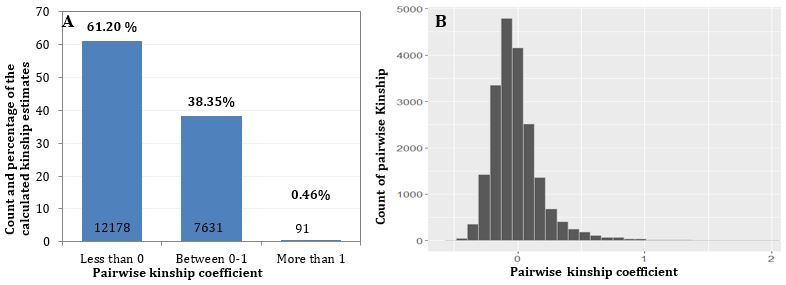

Supplement: Supplementary file 6 — Figure S6. (A) Classification of pairwise relative kinship into 3 classes; (B) Distribution of pairwise relative kinship estimates among 200 wheat cultivars. [file PLD3-6-e438-s008.jpg]

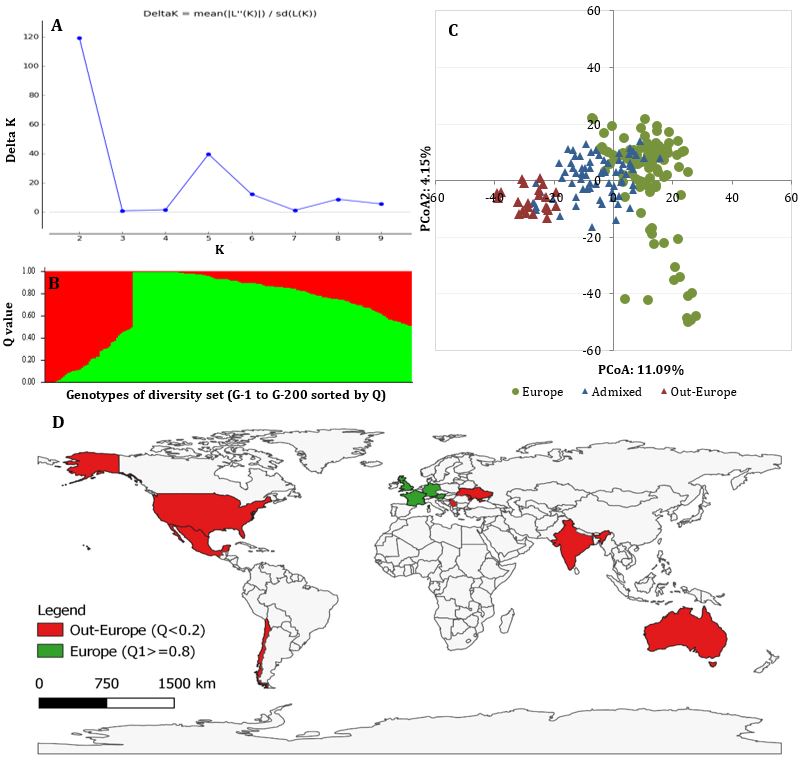

Supplement: Supplementary file 7 — Figure S7. Representation of the wheat panel population structure. [file PLD3-6-e438-s003.jpg]

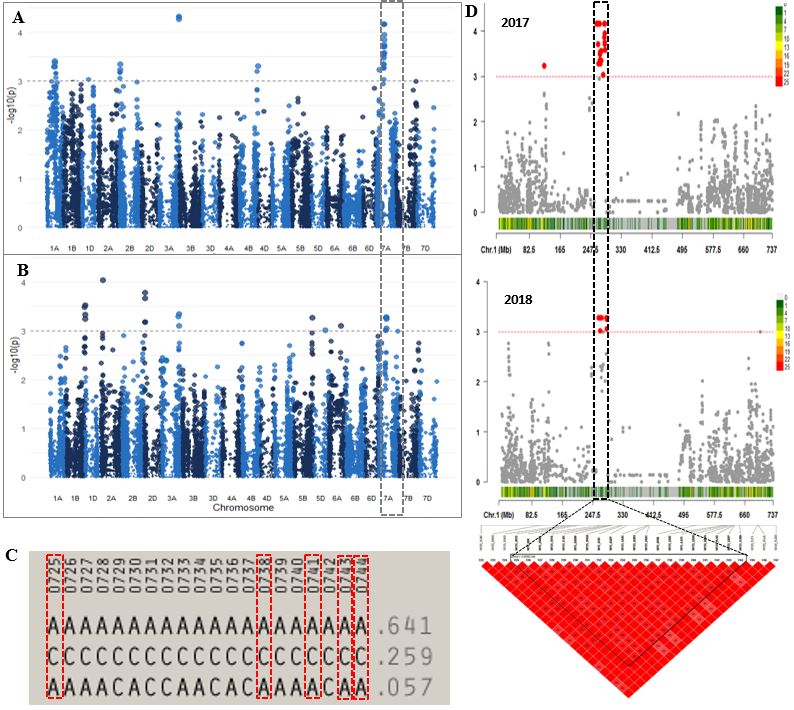

Supplement: Supplementary file 8 — Figure S8. Illustration of marker‐trait association result for SPAD values. [file PLD3-6-e438-s006.jpg]

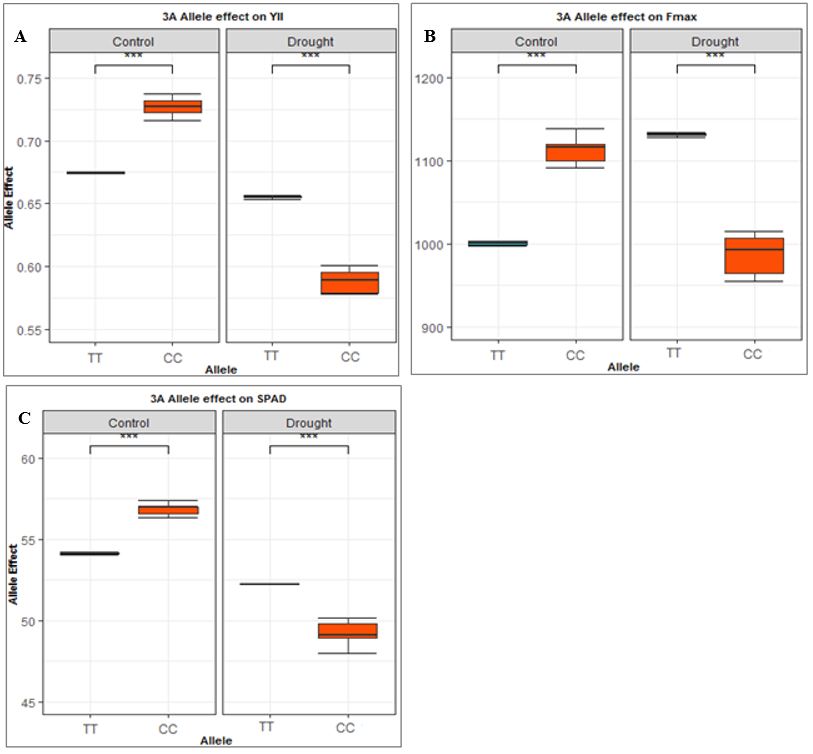

Supplement: Supplementary file 9 — Figure S9. Illustration of marker by treatment interactions on photosynthesis‐related traits [file PLD3-6-e438-s012.jpg]

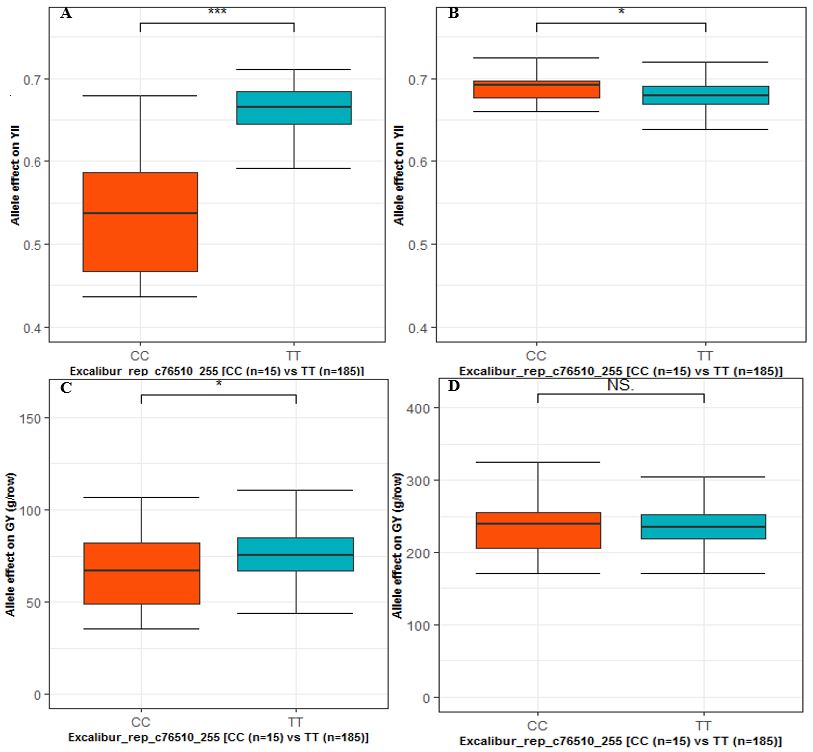

Supplement: Supplementary file 10 — Figure S10. Allelic effect of Excalibur_rep_c76510_255 on YII. [file PLD3-6-e438-s004.jpg]
