# Supplementary material for: Chromosome 3A harbors several pleiotropic and stable drought‐responsive alleles for photosynthetic efficiency selected through wheat breeding
Source: Plant Direct. 2022 Sep 2;6(9):e438. doi: 10.1002/pld3.438 (PMC9440346; doi:10.1002/pld3.438)
Supplement: Supplementary file 12 — Table S1. List of measured leaf chlorophyll a fluorescence parameters. Table S2. Complete description and abbreviation of evaluated traits in the study. Table S3. Chlorophyll content and fluorescence ratio parameters across growth stages in 2017 and 2018 growing seasons. Table S4. Dark and light adapted chlorophyll fluorescence ratio parameters measured at anthesis growth stage Table S5. Stomatal conductance (gsw) dynamic across growth stage in 2018. Table S6. Photosynthesis‐related parameters measured with the Licor 6800 at anthesis growth stage. [file PLD3-6-e438-s009.docx]

# Title: Chromosome 3A harbors several pleiotropic and stable drought-responsive alleles for photosynthetic efficiency selected through breeding of wheat

# Supplementary Tables

## **Table S1 |** List of measured leaf *chlorophyll a* fluorescence parameters

| Extracted Parameter (Abbreviation) | Formula explanation /units | Sample State | Description |
| --- | --- | --- | --- |
| Minimum fluorescence level (F_0_) | Millivolts (mV) | Dark | Measured by very low intensity of measuring light to keep PS II reaction centers open |
| Maximum fluorescence level (F_M_) | Millivolts (mV) | Dark | Measured by a pulse of saturating light (Saturation Pulse) which closes all PS II reaction centers |
| Minimum fluorescence level (F_m_ or F) | Millivolts (mV) | Light | The F corresponds to the momentary fluorescence level (Ft) of an illuminated sample measured shortly before application of a saturation Pulse |
| Maximum fluorescence level (F_max_ or F_M_´) | Millivolts (mV) | Light | The F_M_’ is induced by a Saturation Pulse which temporarily closes all PS II reactions centers. |
| Maximum photochemical quantum yield of PS II (F_V_/F_M)_ | F_V_/F_M_= (F_M_–F_0_)/ F_M_ | Dark | Demonstrates the ability of PSII to perform photochemistry (QA reduction) |
| Effective photochemical quantum yield of PS II (YII) | YII= (F_M_´–F)/ F_M_´ | Light |
| Non photochemical fluorescence quenching (NPQ) | NPQ= (F_M_/F_M_´)-1 | Dark and Light | Estimates the non-photochemical quenching from F_M_ to F_M_´. Monitors the apparent rate constant for heat loss from PSII. |

## **Table S2 |** Complete description and abbreviation of evaluated traits in the study

| Traits | Abbreviation | Descriptions |
| --- | --- | --- |
| *Agronomic traits* | | |
| Grain yield | GY | The plots were harvested and the grains cleaned and weighed and the grain yield in Kg/ha was calculated. |
| Shoot dry weight | SDW | Dry shoot weight (g), dried shoot sample in oven set at 65°C for 3 days and the weight in Kg/ha was calculated |
| Plant biomass weight | PBW | After drying, plant Biomass weight (Biomass) and seed weight was recorded to estimate the HI as the ratio of seed weight to total biomass weight |
| *Developmental traits* | | |
| Relative plant healthiness | HSr | The score of each trait was given with the customized scale from 1 to 5 equivalent to 0, 25, 50, 75 and 100% of damage, where the score 1 was 0 % of bad phenotype indicating the best performance while 100% of bad phenotype was the full expression of worst performance. For example, the leaf greenness was scored as follow 1 means 0 % leaves were yellow and 100% were green; 2 means 25% leaves were yellow, 75% leaves were green and 5 means 100% leaf are yellow. For leaf rolling and greenness, observations were done on the flag leaves and second youngest leaves. |
| Relative plants homogeneity of growth | HGr |
| Relative plant leaves greenness | LGr |
| Relative plant leaves rolling state | LRr |

Table S2 Continues

Table S2 Continued

| *Physiological and functional traits* | | |
| --- | --- | --- |
| Chlorophyll Content | SPAD | SPAD and MINI-PAM measurements were made at the mid-point of a fully expanded leaf, on three plants randomly chosen within a plot using a SPAD 502 instrument (Konica Minolta, Osaka, Japan). During the measurements, special care of leaves angle or shading was observed to avoid change of the ambient state of the leaves (Rascher et al., 2000). The average of the three SPAD measurements per genotype per repetition was calculated and used for the analysis, while the nine data points per genotype from three repetitions were used for analyses. The Chlorophyll a fluorescence parameters evaluated (Walz, 2014) are fully described in Table S1. |
| Effective quantum Yield of Photosystem II | YII |
| Maximum quantum Yield of Photosystem II | F_V_/F_M_ |
| Non-photochemical fluorescence quenching | NPQ |
| Diffusion porometer based Leaf stomatal conductance | LSCp | Diffusion based leaf stomatal conductance (gsLSCp) was measured across four growth stages, prebooting, booting, anthesis and postanthesis by diffusion porometer (AP4-Delta-T Eijelkampt, Giesbech, The Netherlands) with limits operating of 0 to 50 °C temperature and 10 to 90% relative humidity (Devices, n.d.). Readings were done on the second youngest leaves at prebooting and booting, and on flag leaves at anthesis and post-anthesis. Three measurements within a plot of one genotype were made as mmol H2O m-2 s-1 exciting water vapor at full clear air conditions between 10:00 am and 16:30 pm, with about 1500 PAR light intensity and 1000 hPa pressure. |
| Photosynthetic rate | A | Net photosynthetic rate (APR, μmol CO2 m−2 s−1), an IRGA based stomatal conductance (gswLSCl, mol H2O m−2 s−1), intercellular CO2 concentration (CiIntCO2, µmol CO2 mol-1), transpiration rate (ETR, mmol H2O m−2 s−1) and the leaf temperature (T, °C) were investigated using LI-6800 (LI-COR, Lincoln, USA) in open system from 10:00 a.m. to 14:00 a.m. at anthesis growth stage. The photosynthetic active radiation (PAR) of LI-6800 was set as 1000 μmol m−2 s−1. For each line, the flag leaves of three plants in the middle of the plot were selected and the readings were taken at the midpoint part. The difference in temperature (ΔT) was calculated with the formular ΔT=Tleaf- Tair. Leaf instantaneous water use efficiency (LWUE, μmol/ mmol) was calculated as follows LWUE = A/E where A is photosynthetic rate, E is transpiration rate as described by Munjonji et al. (2016). |
| Interceluar CO2 | Ci |
| Transpiration rate | E |
| IRGA based Stomatal conductance | LSCl |
| Leaf instantaneous water use efficiency | LWUE = A/E |
| Difference temperature Leaf-Air | DTLA |

## **Table S3 |** Chlorophyll content and fluorescence parameters of the core set across growth stages in 2017 and 2018 growing seasons and ANOVA significance level of growing seasons and interactions effects

| Physio logical Traits | | Statistic | | Water regime (W) | BBCH growth stage in 2017 | | | | BBCH growth stage in 2018 | | | | | | | Heritability (%) | |  |
| --- | --- | --- | --- | --- | --- | --- | --- | --- | --- | --- | --- | --- | --- | --- | --- | --- | --- | --- |
|  |  |  |  |  | GS40-49 | | GS50-59 | GS60-69 | GS30-39 | GS40-49 | GS50-59 | GS60-69 | | GS70-85 | | H^2^ | h^2^ |  |
| SPAD | | Mean | | Control | 52.72 | | 53.24 | 52.86 |  |  | 54.00 | |  | |  | 92.57 | 47.33 | |
|  |  |  |  | Drought | 52.12 | | 53.24 | 51.03 |  |  | 52.78 |  | |  | | 67.07 | 35.22 |  |
|  |  |  |  | Reduction (%) | 1.13 | | 0.00 | 3.47 |  |  | 2.26 |  | |  | |  |  |  |
|  |  | CV (%) | | Control | 5.95 | | 7.06 | 10.19 |  |  | 7.83 |  | |  | |  |  |  |
|  |  |  |  | Drought | 7.48 | | 6.20 | 14.03 |  |  | 8.50 |  | |  | |  |  |  |
|  |  | Treatment effect | | Water (W) | ** | | ns | * |  |  | ns |  | |  | |  |  |  |
|  |  |  |  | Genotype (G) | *** | | *** | * |  |  | ** |  | |  | |  |  |  |
|  |  |  |  | W*G | * | | *** | ns |  |  | ns |  | |  | |  |  |  |
| YII | | Mean | | Control | 0.61 | | 0.63 | 0.74 | 0.60 | 0.59 | 0.58 | 0.62 | | 0.64 | | 0 | 8.69 | |
|  |  |  |  | Drought | 0.61 | | 0.59 | 0.54 | 0.62 | 0.63 | 0.63 | 0.63 | | 0.56 | | 0 | 6.37 | |
|  |  |  |  | Reduction (%) | 0.00 | | 6.35 | 27.03 | -3.33 | -6.78 | -8.62 | -1.61 | | 12.50 | |  |  | |
|  |  | CV (%) | | Control | 20.60 | | 21.88 | 8.55 | 9.05 | 10.06 | 11.25 | 9.96 | | 12.02 | |  |  | |
|  |  |  |  | Drought | 9.35 | | 23.05 | 34.75 | 11.26 | 9.40 | 9.66 | 8.56 | | 24.53 | |  |  | |
|  |  | Treatment effect | | W | ** | | *** | *** | *** | *** | *** | ns | | *** | |  |  | |
|  |  |  |  | G | *** | | *** | *** | ** | * | ** | *** | | ** | |  |  | |
|  |  |  |  | W*G | * | | *** | *** | *** | ns | * | ns | | ns | |  |  | |
| Growing season effect | | | | | SPAD | | | | YII | | | | | | | | | |
| Growing Year (Y) | |  | | | * | | | *** | | | | | | | | | |  |
| Y*W | |  | | | ns | | | * | | | | | | | | | |  |
| Y*G | |  | | | ns | | | ** | | | | | | | | | |  |
| Y*W*G | |  | | | ns | | | ** | | | | | | | | | |  |

GS30-39: Prebooting growth stage; GS40-49: Booting; GS50-59: S3_Heading; GS60-69: Anthesis, GS70-85: Postanthesis, Fmin, Fmax are respectively the minimum and maximum fluorescence of light acclimated sample; YII is the effective photochemical, Significance levels: *P < 0.05, **P < 0.01, ***P < 0.001, ns means not significant.

## **Table S4 |** Dark and light adapted chlorophyll fluorescence ratio parameters measured at anthesis stage

| Statistic | Water regime (W) | 2017 | | 2018 | |
| --- | --- | --- | --- | --- | --- |
|  |  | Fv/ F_M_ | NPQ | Fv/F_M_ | NPQ |
| Mean | Control | 0.77 | 0.54 | 0.83 | 0.95 |
|  | Drought | 0.75 | 0.26 | 0.80 | 0.49 |
|  | Reduction (%) | 3.25 | 50.77 | 3.56 | 47.90 |
| CV (%) | Control | 3.77 | 46.70 | 1.16 | 31.87 |
|  | Drought | 3.61 | 113.51 | 8.38 | 54.61 |
| Treatment effect | W | *** | *** | *** | *** |
|  | Genotype (G) | ns | * | *** | * |
|  | W*G | ns | *** | ** | *** |
| Growing season effect | | Fv/Fm | | NPQ | |
| Growing Year (Y) |  | *** | | *** | |
| Y*W |  | ns | | * | |
| Y*G |  | ns | | ns | |
| Y*W*G |  | ns | | ns | |

F_Min_, F_Max_ are respectively the minimum and maximum fluorescence of dark adapted sample; Fv/F_Max_ is the maximum photochemical quantum yield of PSII, NPQ in the non-photochemical quenching. Significance levels: *P < 0.05, **P < 0.01, ***P < 0.001, ns means not significant.

## **Table S5 |** Stomatal conductance (gsw) dynamic across growth stage in 2018

| Statistic | Water Regime (W) | Stomatal conductance (gsw) (mol m⁻² s⁻¹) | | | |
| --- | --- | --- | --- | --- | --- |
|  |  | GS30-39 | GS40-49 | GS60-69 | GS70-85 |
| Mean | Control | 0.24 | 0.22 | 0.33 | 0.28 |
|  | Drought | 0.32 | 0.22 | 0.13 | 0.09 |
|  | Reduction (%) | -33.33 | 0.00 | 60.61 | 67.86 |
| CV(%) | Control | 85.45 | 203.70 | 36.21 | 25.28 |
|  | Drought | 156.92 | 60.32 | 44.69 | 77.64 |
| Treatment  effect | W | ns | ns | *** | *** |
|  | Genotype (G) | ns | ns | * | ns |
|  | W*G | *** | ns | ns | ns |

GS30-39: Prebooting growth stage; GS40-49: Booting; GS60-69: Anthesis, GS70-85: Postanthesis,

## **Table S6 |** Photosynthesis-related parameters measured with the Licor 6800 at anthesis growth stage

| Water regime | A (µmol m⁻² s⁻¹) | E (mol m⁻² s⁻¹) |  | LWUE (µmol/mol) | Ci (µmol/mol) | LSCl (mol m⁻² s⁻¹) |
| --- | --- | --- | --- | --- | --- | --- |
| Control | 10.089 | 0.003 |  | 3835.472 | 304.520 | 0.232 |
| Drought | 7.110 | 0.001 |  | 5279.863 | 277.275 | 0.115 |
| Reduction (%) | 29.53 | 66.67 |  | -37.66 | 8.95 | 50.43 |
| Drought effect (P-Value) | 0.007 ** | <0.001 *** |  | 0.011* | 0.034 * | <0.001 *** |

A = Photosynthetic rate; E = Transpiration rate; LWUE = Leaf instantaneous water use efficiency; Ci = Intercellular CO_2_; LSCl = IRGA based Stomatal conductance. Significance levels: *P < 0.05, **P < 0.01, ***P < 0.001, ns means not significant.

## **Table Sxl1 |** Description of SNP number per chromosome and per LD, and chromosomal LD

## **Table Sxl2 |** Number detected significant MTAs (10^-4^) from GWAS under both water regimes in 2017, 2018 and with the mean of both growing seasons

## **Table Sxl3 |** **|** Number detected significant MTAs in the marker*treatment interaction GWAS of three traits Fmax, SPAD, and YII

## **Table Sxl4 |** Table of the significant SNPs in the marker*treatment interaction GWAS

## **Table Sxl5 |** GWAS results from four physiological traits and three final aboveground biomass traits highlighting SNPs with stable and pleiotropic effects

## **Table Sxl6 |** Candidate genes in the QTLs regions with of stability and pleiotropic effects

## **Table Sxl7 |** Candidate genes in the QTLs regions with high significant marker by treatment interactions effects

## **Table Sxl8 |** Table of significant SNPs in the genome wide SNP-SNP epistatic interaction

## **Table Sxl9 |** Candidate genes in the QTLs regions harbouring SNPs with epistatic effects

#
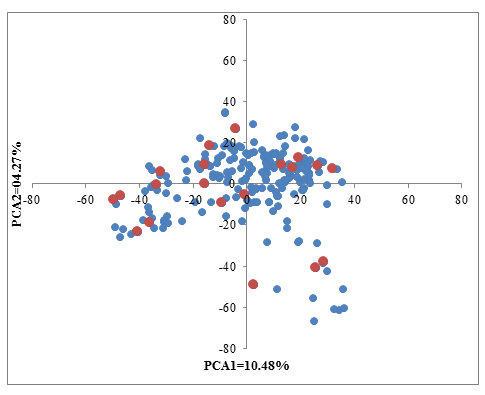
Supplementary Figures

## **FIGURE S1 |** Genetic diversity of 200 winter wheat cultivars (in blue) including the core set of 20 genotypes (in red)

## **FIGURE S2 |** Pearson correlation coefficients between photosynthesis related under rainfed conditions (panel A). Correlation between photosynthesis related traits (grey square) and scored developmental traits under prolonged drought stress conditions (panel B). Lower diagonal panels represents the scatter plot with red line depicting the best fit. The upper panel represents the Pearson correlation coefficient value and size of the correlation coefficient is proportional to the strength of the correlation. The correlation coefficient significance level *P<0.05, **P<0.01, ***P<0.001. The abbreviations of traits names are given in Table S2

**A**

**B**

**B**

**A**

**D**

**C**

## **FIGURE S3 |** Principal component analysis biplot using 11 photosynthesis and transpiration related variables under (A) rainfed, and (C) prolonged drought stress condition. Cosines square of the variables contributing to the newly constructed principal components under rainfed (B) and (D) prolonged drought stress condition. The abbreviations of traits name are found in Table S2.

*Tolerant genotypes*

*Sensitive genotypes*

## **FIGURE S4 |** SNP density across genomes of the studied winter wheat genotypes. (A) the number of SNP on each genome and the total number of SNP. (B) illustrates the number of SNP on each chromosome (from chromosome 1A to 7D). (C) Heatmap of the number of SNPs within 1 Mb window size per chromosome.

**A**

**B**


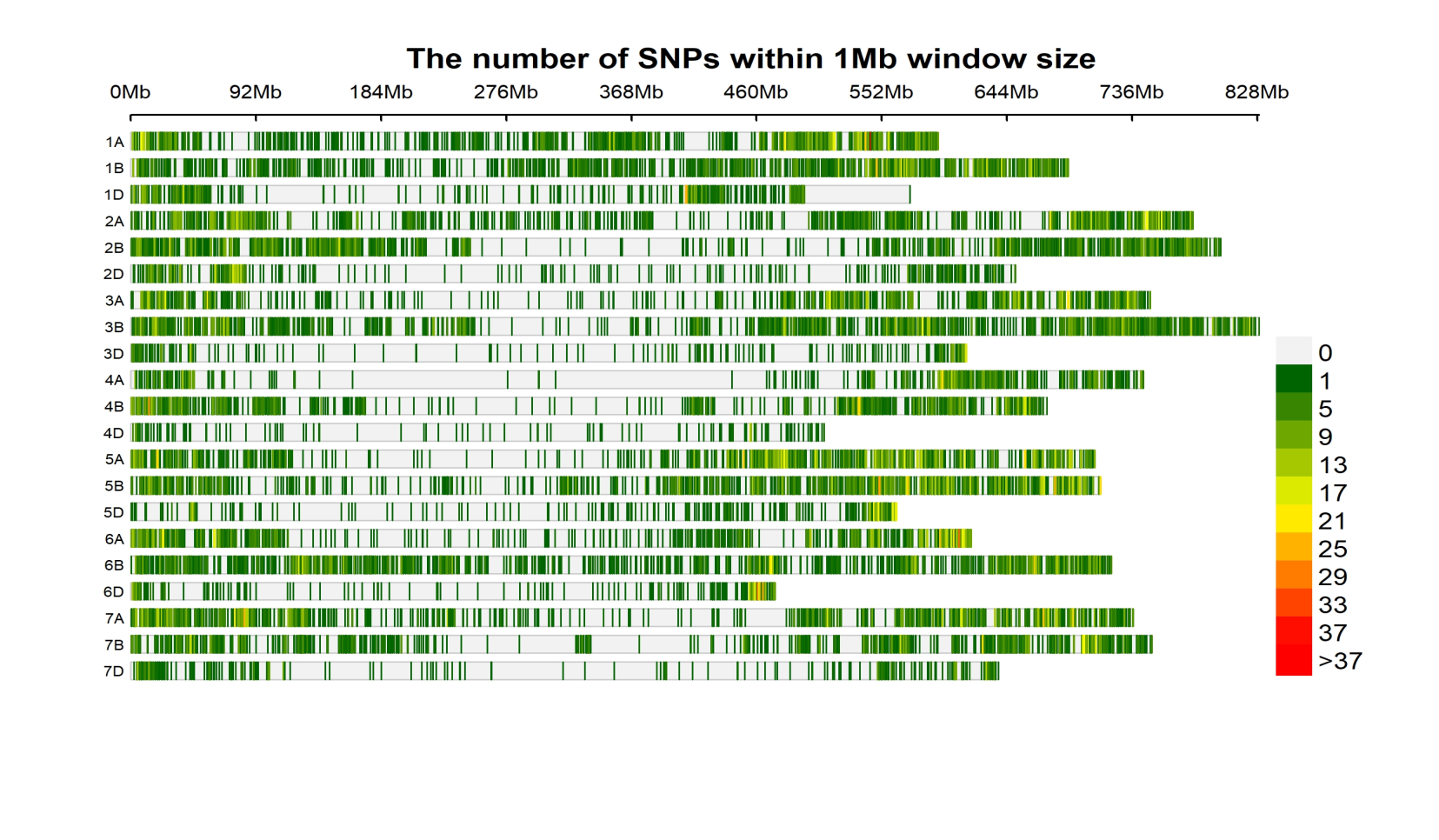


**C**

## **FIGURE S5 |** Sliding windows showing the rate of linkage disequilibrium decay among the 200 genotypes of the diversity set across A, B, D genomes. The last window shows all three genomes in plot (non-fitting curve of genome A, B and D are colored in red, green and blue, respectively). The genetic distance corresponding to r^2^ =0.1 were 19.0, 38.5 and 17.5 Mbp respectively for A, B and D genomes, and was considered as the critical distance up to which a QTL could extend.

**Pairwise kinship coefficient**


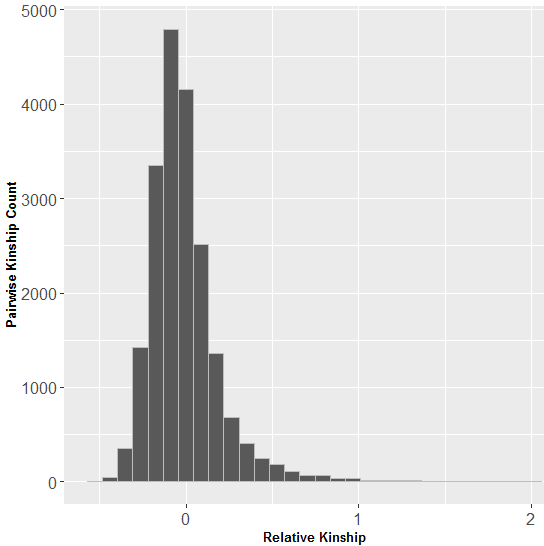


**Count of pairwise Kinship**

**B**


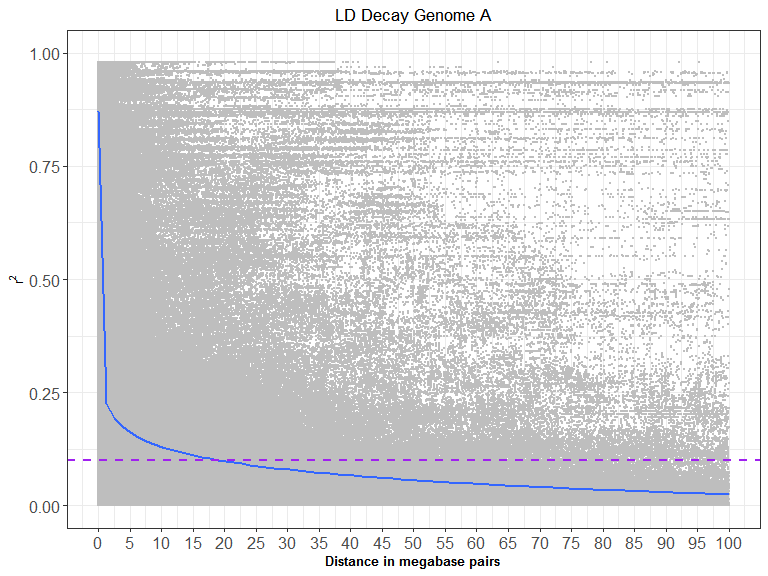

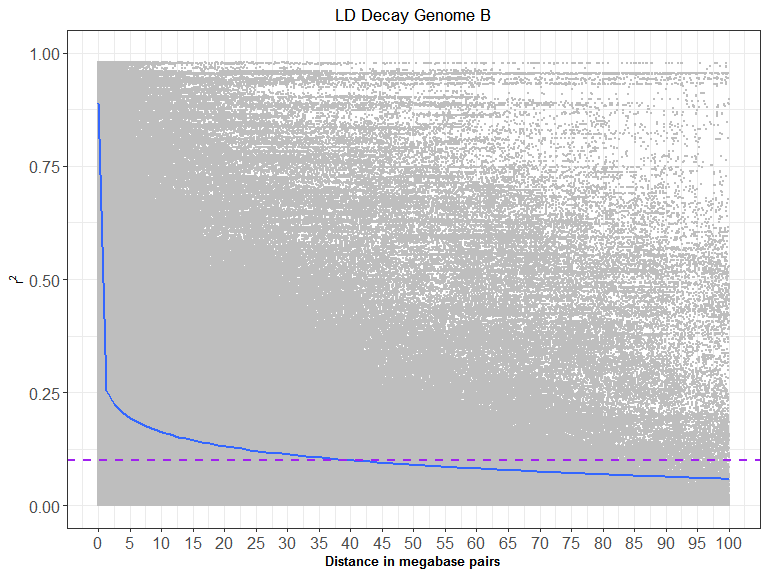

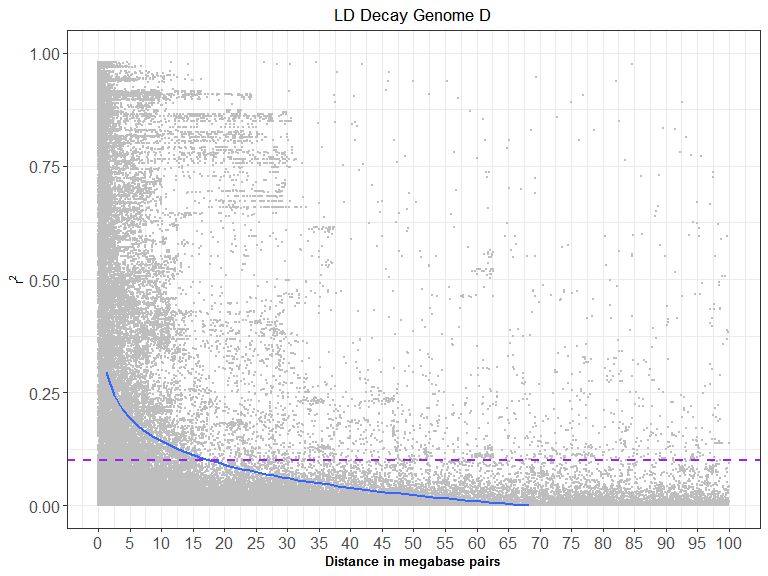

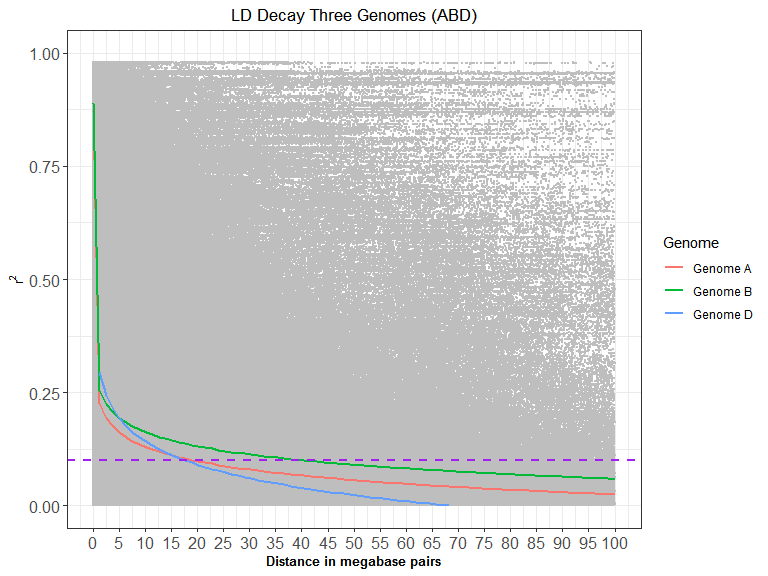


## **FIGURE S6 |** **(A)** Classification of pairwise relative kinship into 3 classes; **(B)** Distribution of pairwise relative kinship estimates among 200 wheat genotypes.

## **FIGURE S7 |** Representation of the wheat panel population structure. **(A)** Inferred population structure based on the change of LnP(D) between consecutive K method developed by Evanno et al. (2005). **(B)** Display of ancestry coefficient Q of two subpopulations at K = 2 from STRUCTURE analysis. **(C)** Principle components analysis (PCA) of individual cultivars of the diversity set. Legend indicates cultivars originated from Europe (99 cultivars in green), out of Europe (25 cultivars in red) and admixed (76 cultivars in blue). **(D)** Geographical representation of ancestry coefficient Q1 at K=2 showing country of origin of the two subpopulations (Europe included Germany, Great Britain, France, and Austria, while Out-Europe comprised USA, Serbia, Ukraine, Australia, Moldavia, Mexico, and Australia).


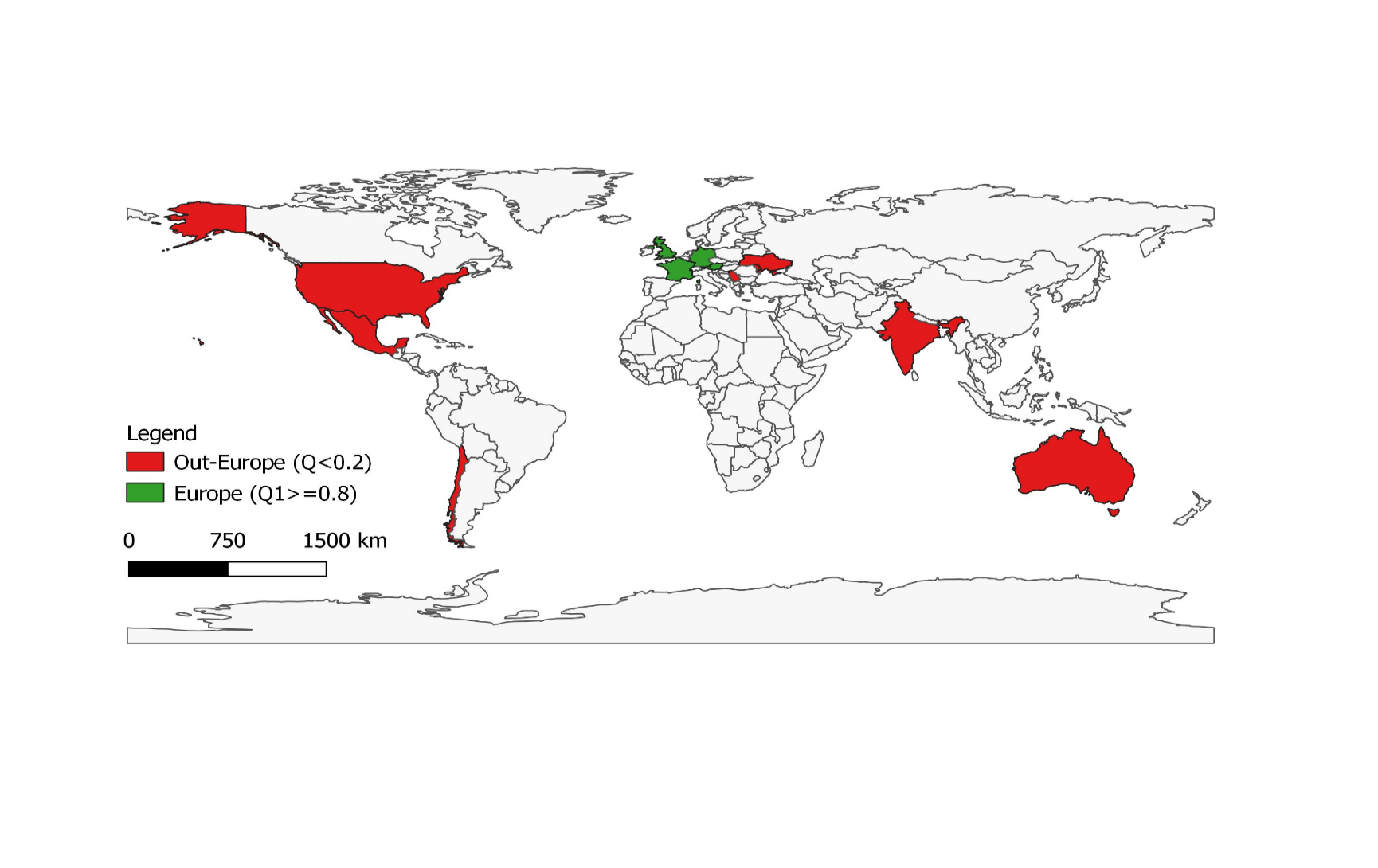


**D**


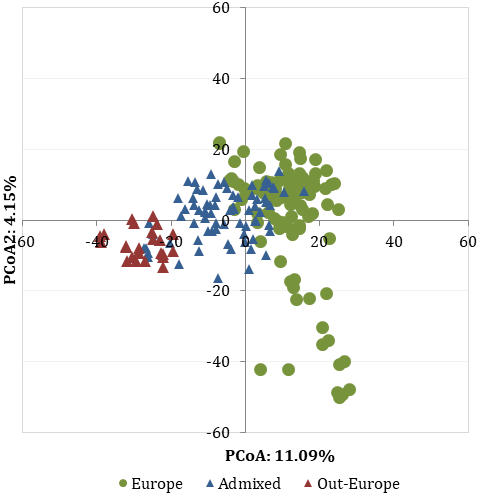


**C**


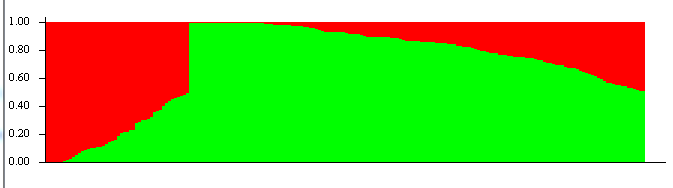


**B**

**Genotypes of diversity set (G-1 to G-200 sorted by Q)**

**Q value**


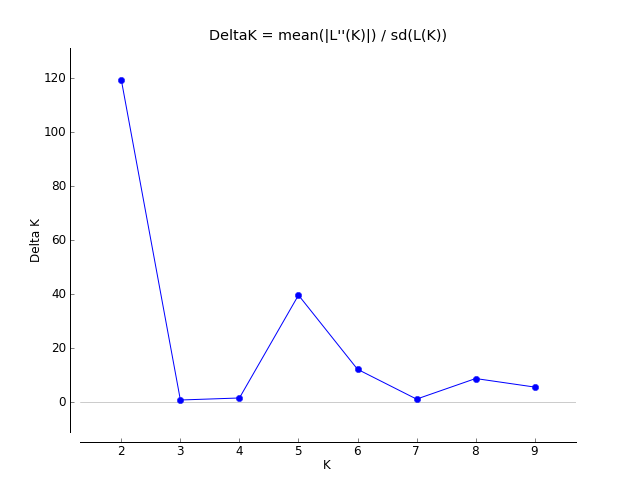


**A**

**Delta K**

**K**

## **FIGURE S8 |** Illustration of marker-trait association result for SPAD. **(A)** Manhattan plot for SPAD under control including significant MTAs in 2017 and **(B)** 2018. (**C**) Haplotype block on chromosome 7A comprising five SNPs [AX-94760655 (725), AX-158600987 (738), AX-108905937 (741), AX-158601006 (743), and AX-158591424 (744)] associated with SPAD in both years. (**D**) Manhattan plot showing a hotspot of 5 stable SNPs of CHR 7A region of 18 Mbp length delimited from AX-94760655 (267.569 Mbp) to AX-158591424 (286.152 Mbp) associated with SPAD in 2017 and 2018.

**D**


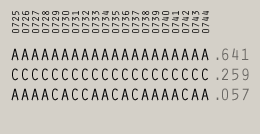


**C**


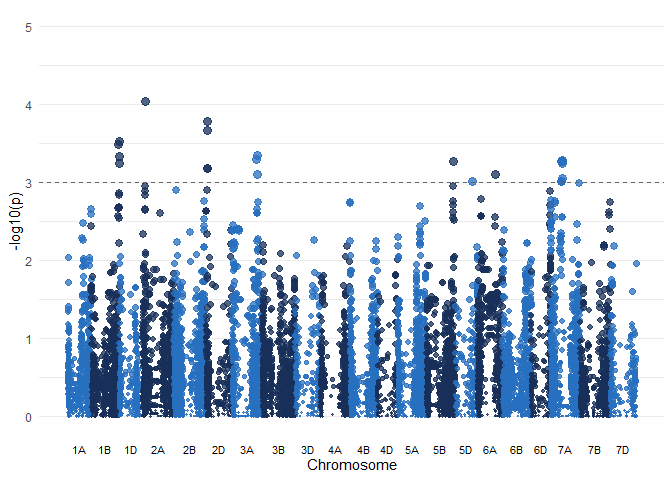

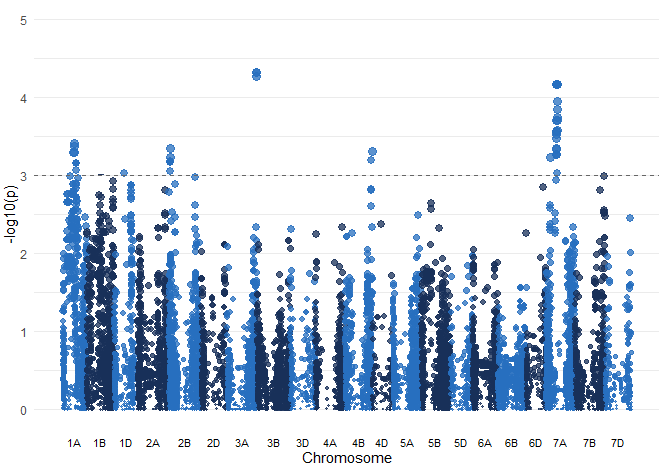


**A**

**B**


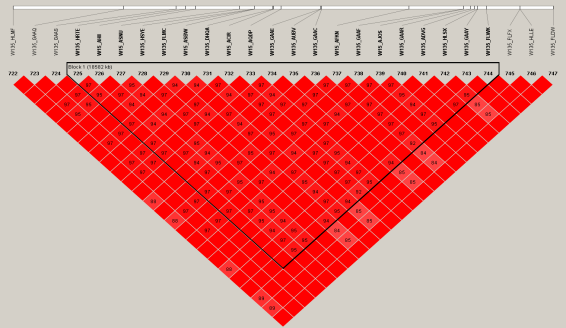

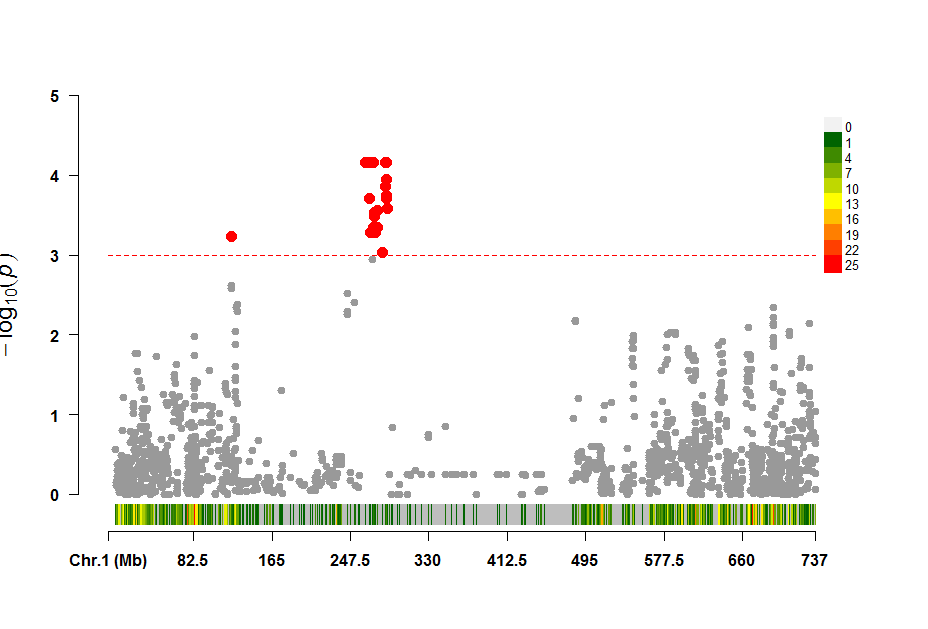

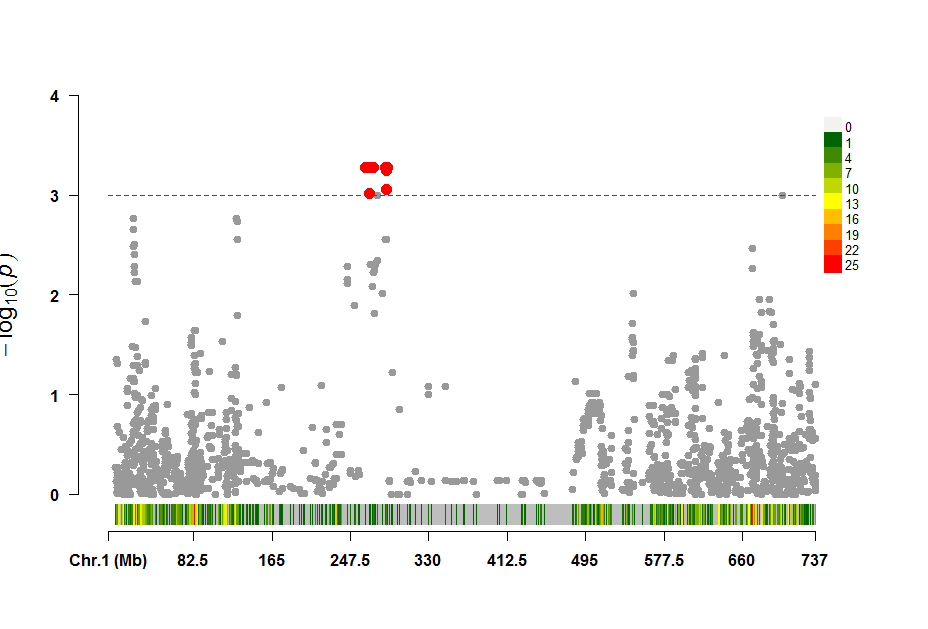


**2017**

**2018**

## **
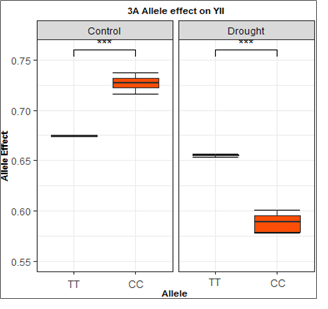

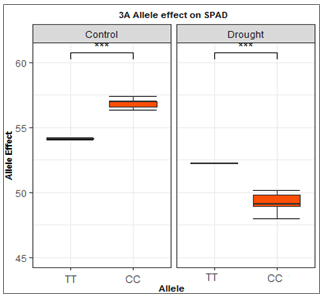

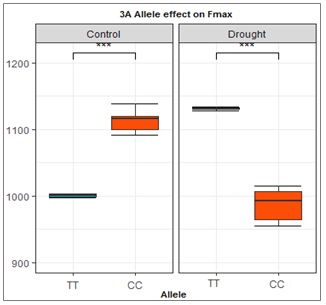
FIGURE S9 |** Illustration of marker by treatment interactions on photosynthesis related traits: (A) effective quantum yield of photosystem II; (B) maximum chlorophyll fluorescence; (C) chlorophyll content. Major alleles (TT) had higher values than the minor alleles CC) under drought conditions whereas the contrary schema was observed in control under rainfed condition.

**C**

**B**

**A**

##
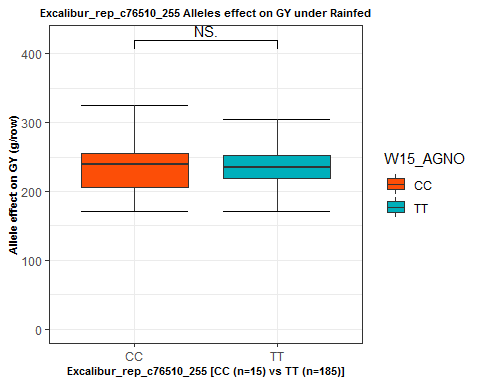

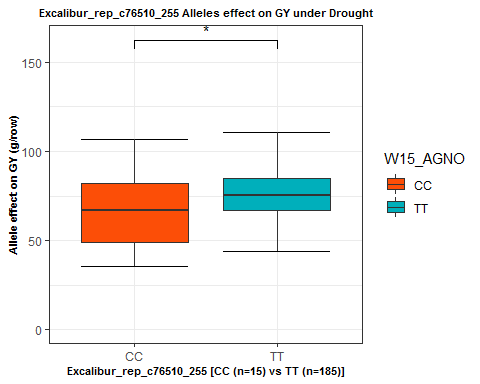

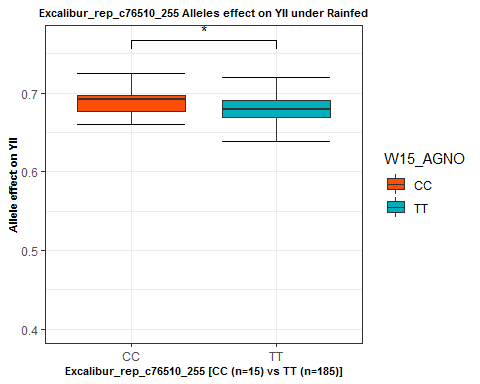

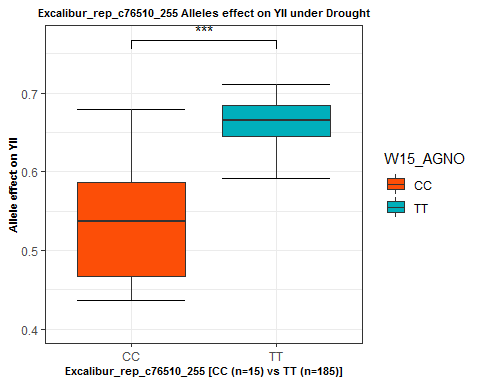
**FIGURE S10** | Allelic effect of Excalibur_rep_c76510_255 on YII under drought (A) and rainfed (B); allelic effect on GY under drought (C) and rainfed (D). Two-sapmle *t-*test P-value shows significant allelic effect difference with reference to major and minor allele.

**C**

**D**

**B**

**A**

**A**

**G**

**F**

**E**
